# RSL3 Promotes STAT3 Ubiquitination to Induce Autophagy and Apoptosis in PARPi-Resistant Breast Cancer Cells

**DOI:** 10.3390/biom15121749

**Published:** 2025-12-18

**Authors:** Lingyan Chen, Dejian Chen, Fengzhuo Yang, Xinyi Chen, Binjiao Zheng

**Affiliations:** 1 School of Nursing, Wenzhou Medical University, Wenzhou 325035, China; 2 Key Laboratory of Laboratory Medicine, Ministry of Education, Wenzhou Key Laboratory of Cancer Pathogenesis and Translation, School of Laboratory Medicine and Life Sciences, Wenzhou Medical University, Wenzhou 325035, China; chendejian@wmu.edu.cn; 3 Key Laboratory of Laboratory Medicine, Ministry of Education, Zhejiang Provincial Key Laboratory of Medical Genetics, School of Laboratory Medicine and Life Sciences, Wenzhou Medical University, Wenzhou 325035, China; fengzhuoyang@wmu.edu.cn (F.Y.); cxy220304@wmu.edu.cn (X.C.)

**Keywords:** RSL3, PARPi-resistant breast cancer, STAT3, ubiquitination, apoptosis

## Abstract

Background: Breast cancer remains the most common malignancy among women worldwide. Current systemic treatment strategies include chemotherapy, immunotherapy, bone-stabilizing agents, endocrine therapy for hormone receptor-positive disease, anti-HER2 therapy for HER2-positive disease, and poly (ADP-ribose) polymerase (PARP) inhibitors for BRCA mutation cases. However, effectively overcoming drug resistance and reducing recurrence and metastasis rates remain major therapeutic challenges. Methods: To investigate the underlying mechanism of RSL3 in PARPi-resistant breast cancer cells, we treated several PARPi-resistant breast cancer cells with varying doses of RSL3. The regulatory proteins of STAT3 were analyzed using real-time quantitative polymerase chain reaction (RT-qPCR) and Western blot analysis. Immunoprecipitation and ubiquitination assay were performed to identify the STAT3 ubiquitination levels. Results: Recently, we identified that RSL3, a ferroptosis activator, exhibits potent antitumor activity against PARPi-resistant breast cancer. Yet, its underlying mechanism remains unclear. Here, we demonstrate that RSL3 directly targets STAT3 and promotes its degradation via the ubiquitination pathway, leading to increased LC3-II levels and decreased p62 expression. These changes ultimately enhance autophagy, which at least partially contributes to elevated apoptosis. Rescue experiments confirmed that STAT3 overexpression reverses RSL3-induced autophagy and apoptosis. Conclusions: Our findings highlight RSL3 as a promising therapeutic agent and STAT3 as a potential target for treating PARPi-resistant breast cancer.

## 1. Introduction

Breast cancer remains a leading cause of cancer-related mortality among women globally [1]. Despite advances in targeted therapies [2,3,4,5], the development of resistance to PARP inhibitors (PARPi) presents a significant clinical hurdle, particularly in BRCA-mutated cancers. Although PARPi exploit synthetic lethality by impairing DNA repair in BRCA-deficient cells [6,7], resistance mechanisms often lead to treatment failure, such as restoration of homologous recombination, drug efflux, and alterations in PARP expression [8]. Therefore, identifying novel strategies to overcome PARPi resistance is urgently needed.

Ferroptosis, an iron-dependent form of programmed cell death driven by lipid peroxidation, has emerged as a promising avenue in cancer therapy. It plays important roles in tumor growth [9,10], neurological disorders [11], and cardiovascular system diseases [12,13]. RSL3, a well-known ferroptosis inducer, acts by inhibiting glutathione peroxidase 4 (GPX4), thereby promoting lipid peroxidation [14]. Beyond ferroptosis, growing evidence suggests that RSL3 can also trigger apoptosis [15]. Recently, we also found that RSL3 could trigger PARP1-mediated apoptosis to inhibit the PARPi-resistant breast cancer growth arrest [16], though the precise mechanisms are not fully understood.

Autophagy, a conserved catabolic process, degrades damaged cellular components via lysosomes to maintain homeostasis. It regulates reactive oxygen species (ROS) generation, iron metabolism, and lipid metabolism, thereby influencing ferroptosis [17,18]. Notably, ferroptosis is considered an autophagy-dependent cell death process [19,20,21]. RSL3 has been shown to induce autophagosome formation and ferritinophagy, enhancing ferroptotic sensitivity [22,23]. Moreover, RSL3 can promote apoptosis in a ROS-dependent manner [15], suggesting intricate crosstalk among ferroptosis, autophagy, and apoptosis.

Signal transducers and activators of transcription 3 (STAT3), a transcription factor and signal transducer, is frequently activated in cancers and promotes tumor survival, proliferation, and anti-apoptotic signaling. Cytoplasmic STAT3 also suppresses autophagy, and its degradation can induce lysosomal membrane permeabilization and apoptosis [21,24,25]. Previous studies, including our own, have shown that STAT3 degradation via the ubiquitin–proteasome pathway decreases p62 level and increases LC3-II level to promote autophagic cell death [26,27]. Importantly, we have already demonstrated that genetic mutation of STAT3 led to a significant increase in LC3-II accumulation and endogenous puncta levels, indicating enhanced autophagic flux [27]. However, whether RSL3 modulates STAT3 to regulate autophagy and apoptosis in PARPi-resistant breast cancer has not been explored.

In this study, we investigated the molecular mechanisms by which RSL3 exerts its anti-tumor effects in PARPi-resistant breast cancer cells. We found that RSL3 directly binds to STAT3 and promotes its ubiquitination and degradation, leading to enhanced autophagy and apoptosis. Overexpression of STAT3 reversed these effects, confirming its central role in RSL3-induced cell death. Our results reveal a novel mechanism by which RSL3 induces autophagy and apoptosis via STAT3 ubiquitination, offering new insights for overcoming PARPi resistance.

## 2. Materials and Methods

### 2.1. Cell Lines and Cell Culture

Human HCC1937 cells were obtained from Guangzhou Xinyuan Technology Co., Ltd. (Guangzhou, China) Human MCF7, MDA-MB-453, and HEK293T cells were procured from the Cell Bank of the Chinese Academy of Sciences (Shanghai, China). Human MDA-MB-453, MCF-7, and HEK293T cells were cultured in Dulbecco’s modified Eagle’s medium (DMEM, Gibco, Thermo Fisher Scientific, Waltham, MA, USA). Human HCC1937 cells were cultured in RPMI-1640 medium (Gibco, Thermo Fisher Scientific, MA, USA). All cells were cultured in culture media supplemented with 10% fetal bovine serum (FBS, Newzerum, Christchurch, New Zealand) and 1% penicillin-streptomycin (Gibco, Thermo Fisher Scientific, MA, USA) at 37 °C in a humidified incubator with 5% CO_2_. Cells were authenticated by short tandem repeats (STR) and routinely checked and confirmed to be free of mycoplasma.

To establish PARPi-resistant cell lines, MDA-MB-453 and HCC1937 cells were continuously exposed to increasing concentration of Olaparib (MedChemExpress, Monmouth Junction, NJ, USA) over a period of 6 months, starting from 0.1 µM up to 10 µM. The resistant cells were maintained in medium containing 1 µM Olaparib and were confirmed to exhibit significantly reduced sensitivity to Olaprib compared to their parental counterparts, as assessed by cell viability assays.

### 2.2. Reagents and Antibodies

FITC Annexin V Apoptosis Detection Kit I was from BD Pharmingen^TM^ (Franklin Lakes, NJ, USA). RSL3, (BafA1), chloroquine (CQ), 3-Methyladenine (3-MA), Z-Leu-Leu-Leu-al (MG132), ferrostatin-1, and Liproxstatin-1 (Lip-1) were purchased from MedChemExpress (Monmouth Junction, NJ, USA). Pierce BCA^TM^ Protein Assay Kit was purchased from the Beyotime Institute of Biotechnology (Haimen, Jiangsu, China). Cycloheximide (CHX) was obtained from Aladdin Biochemical Technology Co., Ltd. (Shanghai, China). Horseradish peroxidase (HRP)-conjugated secondary antibodies (goat anti-rabbit and goat anti-mouse) were purchased from the Beyotime Institute of Biotechnology (Haimen, Jiangsu, China).

The primary antibodies used were as follows. anti-p62 (ab56416), and anti-LC3B (ab192890) were purchased from Abcam (Cambridge, UK). Anti-STAT3 (10253-2-AP) was purchased from ProteinTech (Wuhan, Hubei, China). Anti-ubiquitin (sc-8017) was purchased from Santa Cruz Biotechnology (Dallas, TX, USA). Anti-p-STAT3 (Tyr705) (9145S), p-STAT3 (Ser727) (9134S), and STAT3 (12640S) were purchased from Cell Signaling Technology (Beverley, MA, USA). Anti-β-actin (GB15003) was purchased from Servicebio (Wuhan, Hubei, China).

### 2.3. Apoptosis Analysis

MDA-MB-453, MCF7, and HCC1937 cells were seeded into 6-well plates at a density of 6 × 10^4^ cells/per well, incubated overnight at 37 °C in an atmosphere containing 5% CO_2_, and then treated with the indicated concentration of RSL3 for 12 h. Cells were collected, washed with pre-cold 1 × PBS, and resuspended in 1 × binding buffer, followed by incubating with Annexin V-FITC/PI in the dark at room temperature for 15 min. Stained cells were measured on a BD Accuri^TM^ C6 plus flow cytometer (Franklin Lakes, NJ, USA). All analyses were performed at least 3 times, with a minimum of 20,000 cells per condition.

### 2.4. Western Blotting Analysis

Cells and tissues were lysed in Triton X-100 cell lysis buffer containing phosphate inhibitor cocktail (APExBIO, Houston, TX, USA) for 20 min on ice, and then centrifuged at 12,000 rpm for 20 min at 4 °C. Aliquots of whole cell lysate (15 μg/lane) were separated by 10% to 12% SDS polyacrylamide gels and transferred onto a polyvinylidene fluoride (PVDF) membrane (Millipore, Darmstadt, Germany) in Tris-glycine buffer. The blots were blocked at room temperature for 2 h in 5% non-fat milk in 1 × TBST on a shake and incubated with primary antibodies overnight at 4 °C. The membrane was incubated with HRP-conjugated secondary antibodies at room temperature for 60 min. HRP-bound proteins were detected with enhanced chemiluminescence reagents (ECL, Millipore, Darmstadt, Germany). Images were scanned by ChemiDoc Imaging Systems (Bio-Rad, Irvine, CA, USA) and quantified by using ImageJ 6.1 software.

### 2.5. Immunoprecipitation and Ubiquitination Assay

Immunoprecipitation and ubiquitination assay were performed using a protocol as described previously [28].

### 2.6. RNA Extraction and RT-qPCR

RNA extraction and RT-qPCR were performed using a protocol as described previously [27]. Primers for gene amplification are shown in Table S1.

### 2.7. Immunofluorescence Staining

For the detection of endogenous LC3 puncta, immunofluorescence staining was performed using anti-LC3 antibody to reflect the basal autophagic activity under physiological conditions. MDA-MB-453, MCF-7, and HCC1937 cells were grown on microscope-cover glasses in 24-well plates. After being treated with the indicated dose of RSL3, the cells were fixed with 4% paraformaldehyde for 15 min at room temperature after appropriate treatment. The cells were washed with pre-cold 1 × PBS and rotated gently. The cells were then permeabilized cells with 0.3% Triton X-100 in 1 × PBS for 30 min at room temperature, blocked with blocking buffer (5% BSA and 0.3% Triton-X in PBS) for 2 h at room temperature, and incubated with anti-LC3 (1:1000) for autophagic analysis and anti-STAT3 antibody (1:300) for STAT3 localization analysis. at 4 °C overnight. The cells were washed with 0.3% Triton-X in 1 × PBS and, respectively, incubated with Alexa Fluor Plus 555 (Cell Signaling Technology, Beverley, MA, USA) for 1 h at room temperature. The cells were stained with 4′,6-diamidino-2-phenylindole (DAPI) (Beyotime, Haimen, Jiangsu, China) before mounting and imaging on an LSM900 Zeiss laser confocal microscope (Zeiss, Jena, German).

### 2.8. Constructing Overexpression Cell Lines

Plasmids containing the cDNA of RFP-LC3, GFP-STAT3, and the empty GFP vector (pLVX-Puro vector) were obtained from Miaoling Biotechnology Co., Ltd. (Wuhan, China). Lentiviruses were prepared in 60 mm dishes by transfecting HEK293T cells with 2 µg of plasmid per dish using PEI (Maokang Biotechnology, Shanghai, China). The resulting lentiviruses were used to transfect MCF7, MDA-MB-453, and HCC1937 cells for 48 h. After treatment with 0.5–1.5 µg/mL puromycin (Amresco, Shanghai, China) for 5–7 days, stable cell lines were selected and checked using immunoblotting. To dynamically monitor autophagy and STAT3 localization, cells were co-transfected with RFP-LC3 and either the empty GFP vector (Vector group) or the GFP-STAT3 fusion plasmid (*STAT3* OE group). After treatment with RSL3 for 12 h, live-cell fluorescence imaging was performed directly without antibody staining to visualize RFP-LC3 puncta and GFP-STAT3 signals.

### 2.9. Statistical Analysis

All statistical analysis and graphs were performed using GraphPad Prism 8.0 software (GraphPad Software Inc., San Diego, CA, USA). Data were analyzed using One-way analysis of variance (ANOVA) with Bonferroni post-test, two-way ANOVA with Tukey’s post-test, and two-tailed unpaired Student’s *t*-test. *p* value of less than 0.05 was considered statistically significant (* *p* < 0.05, ** *p* < 0.01, *** *p* < 0.001, **** *p* < 0.0001; n.s., no significant difference). Data were repeated at least three times and expressed as mean ± standard deviation (SD). All quantitative experiments were performed with at least three independent biological replicates to ensure statistical reliability. Quantitation values below the blots correspond to the representative image shown and are normalized to β-actin.

### 2.10. Bioinformatic Analysis

**Figure 1:**
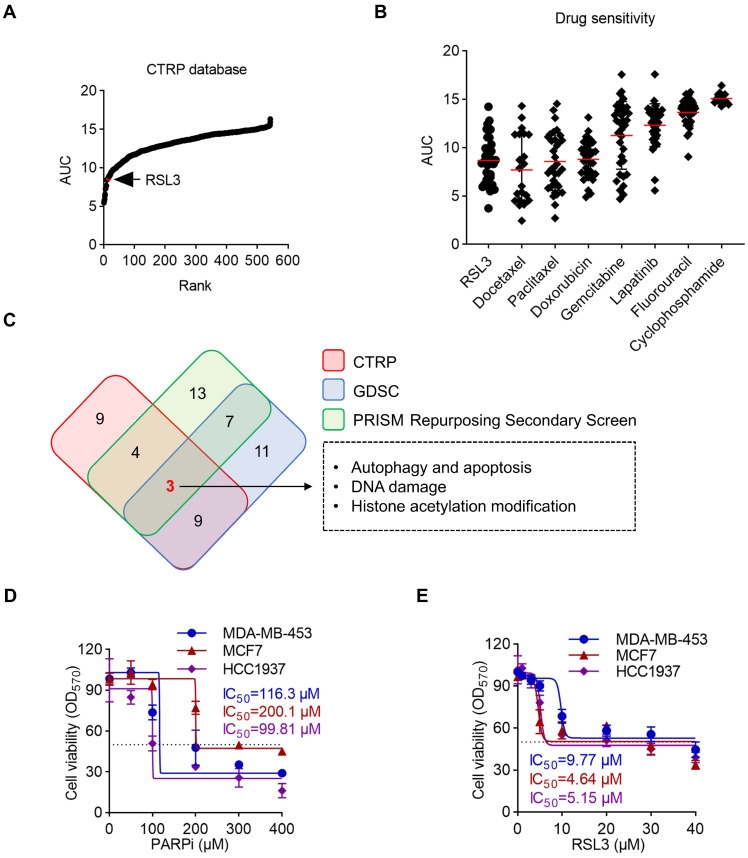
RSL3 is a potential inhibitor for breast cancer treatment. (**A**) Drug sensitivity ranking of 545 compounds across breast cancer cell lines from the CTRP database. Each dot represents a drug, ranked by mean AUC. RSL3 is highlighted (rank 15). (**B**) Comparison of AUC values between RSL3 and clinically used breast cancer drugs across breast cancer cell lines. Each point represents a cell line. (**C**) Venn diagram showing functional overlap among the top 30 drugs from CTRP, GDSC, and PRISM databases. Common mechanisms include autophagy/apoptosis induction, DNA damage response, and histone acetylation regulation. (**D**) Cell viability of PARPi-resistant MDA-MB-453, MCF7, and HCC1937 cells treated with a serial dose of olaparib for 12 h were evaluated using MTT cellular proliferation and cytotoxicity assay kits (*n* = 6). (**E**) IC_50_ values were determined for PARPi-resistant breast cancer cell lines treated with increasing doses of RSL3 for 12 h using MTT cellular proliferation and cytotoxicity assay kits (*n* = 6).

Drug sensitivity data (AUC values) for RSL3 and clinical breast cancer drugs were obtained from the Cancer Therapeutics Response Portal (CTRP) via the DepMap database (https://depmap.org/portal/, accessed on date 15 November 2024). The AUC values were averaged across breast cancer cell lines for each drug. Drugs were ranked based on average AUC, with lower values indicating higher sensitivity. For Figure 1B, AUC values for individual breast cancer cell lines treated with RSL3 or reference drugs were plotted. For Figure 1C, the top 30 drugs from CTRP, GDSC, and PRISM databases were analyzed for functional overlap using Venn diagrams and functional annotation.

## 3. Results

### 3.1. RSL3 Is a Potential Inhibitor for Breast Cancer Treatment

To investigate RSL3′s effect on breast cancer, we analyzed its drug sensitivity in the Cancer Therapeutics Response Portal (CTRP) drug sensitivity database. We found that RSL3 ranked 15th among 545 compounds, suggesting its potent anti-breast cancer activity (Figure 1A). Importantly, RSL3 has a strong antitumor activity compared with the drugs for breast cancer treatment in a clinic, such as docetaxel, paclitaxel, doxorubicin, etc (Figure 1B). The comparison clearly demonstrates that RSL3 exhibits superior cytotoxicity in breast cancer cell lines. How does RSL3 exert its antitumor role? We compared the top 30 drugs from different databases including CTRP, Genomics of Drug Sensitivity in Cancer (GDSC), and PRISM Repurposing Secondary Screen database. We then performed a functional enrichment analysis of their targets. The Venn diagram highlights that the most common mechanisms among these drugs, including autophagy and apoptosis, DNA damage, and histone acetylation modification (Figure 1C). Previous studies found that RSL3 could trigger apoptosis and DNA damage response in human cancer cells [15,29]; however, whether RSL3 induces autophagy in breast cancer cells remains unknown. To confirm the PARPi-resistant phenotype of the cell lines used in this study, we chose MCF7 as a negative control cell line for PARPi resistance [30] and established PARPi-sensitivity cell lines MDA-MB-453 [30] and HCC1937 [31,32] as PARPi-resistant models through prolonged exposure to increasing concentrations of Olaparib. We normalized the OD_570_ value to 100% cell viability using a normalization formula to calculate the IC_50_ value of PARPi and RSL3: (OD_Treatment_ − OD _Blank_/OD_Control_ − OD_blank_) × 100. Dose–response curves were analyzed using Graphpad Prism with nonlinear regression. IC_50_ values represent drug concentrations achieving 50% inhibition of maximal proliferation capacity. Our results showed that the established resistant cells exhibited significantly higher viability, confirming their resistance to PARPi (Figure 1D). To examine the role of RSL3 on PARPi-resistant breast cancer cells, these cells treated with a serial dilution of RSL3 for 12 h and though IC_50_ values ranged from 4.64 to 9.77 µM (Figure 1E).

### 3.2. RSL3 Triggers Excessive Autophagy and Apoptosis

**Figure 2:**
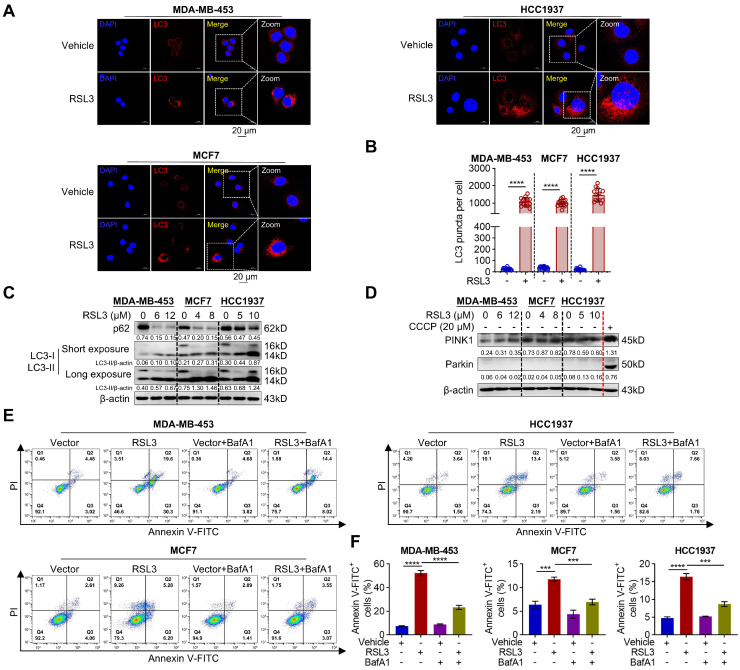
RSL3 induces autophagy and apoptosis in PARPi-resistant breast cancer cells. (**A**,**B**) The endogenous LC3 puncta treated with RSL3 at the indicated concentration (MDA-MB-453: 6 µM; MCF7: 4 µM; HCC1937: 5 µM) for 12 h (**A**) (Scale bar, 20 µm). The ratio of LC3 puncta per cell was quantified using ImageJ Plus software (**B**). Data were shown as mean ± SD, and statistical significance was assessed by two-tailed unpaired Student’s *t*-test (*n* > 3, **** *p* < 0.0001). (**C**) Western blotting analysis of p62 and LC3 treated with a gradient concentration of RSL3 for 24 h. (**D**) Western blotting analysis of mitophagy-related protein PINK1 and Parkin with or without RSL3 before being treated with CCCP (20 µM) for 20 min. (**E**,**F**) Cells were co-treated with RSL3 and BafA1 (25 nM) at the indicated concentration as same as graph (**A**) for 24 h and then stained with annexin V-FITC/PI were determined using flow cytometry (**E**). The apoptosis rate was plotted followed by statistical analysis (**F**). Data were shown as mean ± SD, and statistical significance was assessed by one-way ANOVA with Bonferroni post-test (*n* = 3, *** *p* < 0.001, **** *p* < 0.0001). Original images can be found at Supplementary Materials.

To further confirm that RSL3 induces autophagy and apoptosis, we treated these PARPi-resistant cells with RSL3 at the specified concentration according to their respective IC_50_ values (Figure 1E) and our previous study [16]. Endogenous LC3 staining was used to assess basal autophagy, whereas RFP-LC3 transfection was employed in subsequent rescue experiments to enable precise quantification in genetically modified cells. We found that RSL3-treated cells contained abundant endogenous LC3 puncta compared to the control treatment (Figure 2A,B), implying the formation of autophagic vesicles. To further verify whether RSL3 facilitated autophagy, we examined the levels of autophagy-related proteins. Our findings showed that RSL3 significantly decreased p62, a well-known autophagic substrate, but increased LC3-II/β-actin ratio, indicating enhanced autophagy (Figure 2C). However, mitophagy-related proteins such as PTEN-induced putative kinase 1 (PINK1) and Parkin were unchanged in RSL3-treated PARPi-resistant breast cancer cells (Figure 2D), indicating that RSL3 may induce non-selective autophagy, but not selective autophagy such as mitophagy. Appropriate mitophagy can eliminate damaged cell organelle promptly and is essential for maintaining healthy functions [33]. However, excessive or prolonged autophagy activation eventually induces programmed cell death, such as apoptosis [28,33]. We then examined whether RSL3-triggered autophagy was a protective response or nonapoptotic cell death and observed that bafilomycin A1 (BafA1), an autophagy inhibitor, significantly decreased RSL3-induced apoptosis (Figure 2E, F). These data suggest that RSL3 triggers autophagy and apoptosis in PARPi-resistant breast cancer cells.

### 3.3. RSL3 Directly Targets STAT3 to Decrease Its Protein Levels

**Figure 3:**
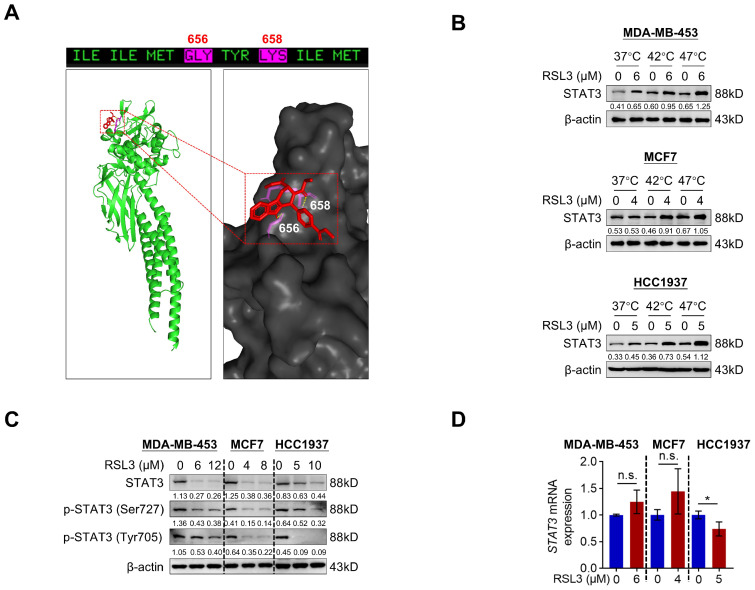
RSL3 directly targets STAT3 to reduce its protein levels. (**A**) The proposed binding models of RSL3 with STAT3 from AlphaFold3 analysis. (**B**) The degradation of STAT3 protein in cells treated with RSL3 for 2 h and then heated at 37 °C, 42 °C, and 47 °C. (**C**) Western blotting analysis of STAT3, p-STAT3 (Tyr705), p-STAT3 (Ser727), p63, and LC3 in Olaparib-resistant cells treated with a gradient concentration of RSL3 for 12 h. (**D**) RT-qPCR analysis of *STAT3* mRNA expression in Olaparib-resistant cells treated with the indicated dose of RSL3 for 12 h. Data were shown as mean ± SD, and statistical significance was assessed by two-tailed unpaired Student’s *t*-test (*n* = 3, * *p* < 0.05 or n.s., not significant by unpaired Student’s *t* test). Original images can be found at Supplementary Materials.

STAT3 is a signal transducer and transcription activator, and it was previously identified as a negative regulator of autophagy in our research [27]. To investigate whether RSL3 targets STAT3, we performed an AlphaFold 3.0 server to conduct a molecular docking analysis and observed that RSL3 had a good docking level with STAT3 through hydrogen bonding with Gly656 and Lys658 (Figure 3A). Meanwhile, cellular thermal shift assay (CETSA) result showed that RSL3 protected STAT3 from thermal degradation, indicating that RSL3 directly targets STAT3 protein (Figure 3B). To identify the regulatory role of RSL3 on STAT3 expression, we treated PARPi-resistant breast cancer cells with a serial dose of RSL3. As shown in Figure 3C, RSL3 dramatically reduced total STAT3, p-STAT3 (Tyr705), and p-STAT3 (Ser727) protein levels without a dramatic effect on *STAT3* mRNA levels, implying that RSL3 may regulate STAT3 expression at the post-transcriptional level (Figure 3C,D). These results suggest that RSL3 can directly target STAT3 to reduce its protein levels.

### 3.4. RSL3 Degrades STAT3 Proteins Through the Ubiquitination Pathway

**Figure 4:**
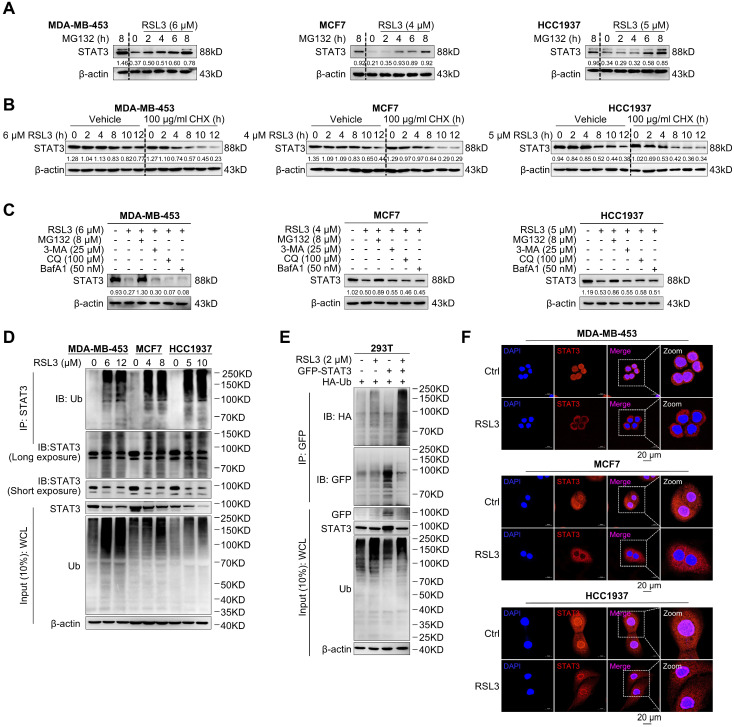
RSL3 degrades STAT3 through ubiquitination pathway. (**A**) Western blot analysis of STAT3 expression in PARPi-resistant breast cancer cells treated with RSL3, the indicated concentrations MG132 (8 µM) alone, or their combination for different times. MG132-only groups are included to control for proteasomal inhibition effects. (**B**) Western blotting analysis of STAT3 expression in cells treated with RSL3 alone or in combination with CHX (100 µg/mL) for the indicated time points. (**C**) Western blotting analysis of STAT3 expression in cells co-treated with RSL3 and MG132, or three autophagy inhibitors 3-MA, CQ, or BafA1 for 12 h. (**D**) Endogenous STAT3 ubiquitination was measured under a serial dose of RSL3 after immunoprecipitation with an antibody against STAT3 and immunoblotted for ubiquitination. (**E**) GFP-STAT3 and hemagglutinin (HA)-ubiquitin were co-transfected into HEK293T cells and analyzed for exogenous STAT3 ubiquitination in cells with or without RSL3 for 12 h. (**F**) Immunofluorescence images of STAT3 (red) and DAPI (blue) in cells treated with RSL3 (MDA-MB-453: 6 µM; MCF7: 4 µM; HCC1937: 5 µM) for 12 h (Scale bar, 20 µm). Original images can be found at Supplementary Materials.

The ubiquitin–proteasome pathway is mainly responsible for STAT3 degradation [26,27], and we thus treated cells with RSL3 and MG132, a proteasome inhibitor. MG132 indeed reversed RSL3-induced reduction in STAT3 protein levels in a time-dependent manner (Figure 4A). Additionally, we treated cells with RSL3 alone or in combination with CHX, an inhibitor of protein synthesis. The combination led to a more rapid decline in STAT3 levels compared to RSL3 alone (Figure 4B), indicating that RSL3 promotes STAT3 degradation and that this effect is enhanced when new protein synthesis is suppressed (Figure 4B). We also treated PARPi-resistant cells with autophagy inhibitors, including 3-MA, CQ, and BafA1 and they failed to block RSL3-induced STAT3 degradation (Figure 4C). Notably, our previous study demonstrated that treatment with 3-MA, CQ, or BafA1 alone did not affect STAT3 protein levels [27], further confirming that RSL3-induced STAT3 degradation is independent of autophagy inhibition. Importantly, RSL3 dramatically increased endogenous and exogenous STAT3 ubiquitination levels (Figure 4D,E). In Figure 4E, the input data show that RSL3 treatment reduces GFP-STAT3 levels but has minimal effect on endogenous STAT3 in non-transfected cells. This indicates that the overexpressed GFP-STAT3 is more susceptible to RSL3-induced ubiquitination and degradation in HEK293T cells. To determine whether RSL3 affects STAT3 subcellular localization, we performed immunofluorescence staining in PARPi-resistant cells. As shown in Figure 4F, RSL3 treatment significantly promotes cytoplasmic translocation of STAT3, although both cytoplasmic and nuclear STAT3 levels were decreased. This is consistent with the role of cytoplasmic STAT3 in autophagy [26] and supports its involvement in RSL3-induced autophagy and apoptosis. These findings indicate that RSL3 promotes STAT3 degradation via the ubiquitin–proteasome pathway.

### 3.5. STAT3 Is an Efficacious Target of RSL3-Mediated Autophagy

**Figure 5:**
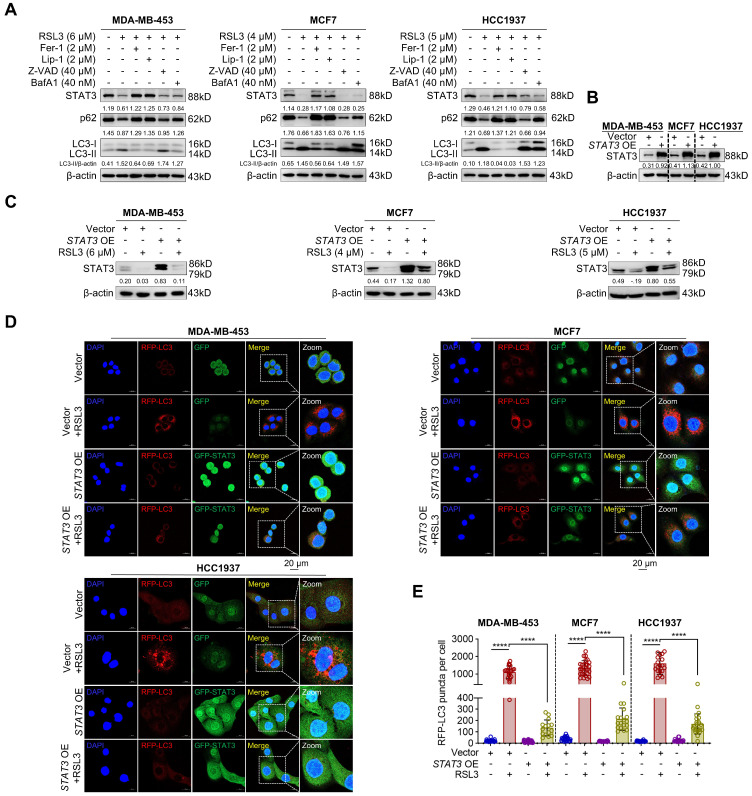
STAT3 is critical for RSL3-induced autophagy. (**A**) Western blotting analysis of STAT3, LC3, and p62 expression in cells co-treated with RSL3 and Fer-1, Lip-1, Z-VAD, or BafA1 for 12 h. (**B**) Western blotting analysis of demonstrating STAT3 overexpression in cells. (**C**) Western blotting analysis demonstrating STAT3 overexpression in the PARPi-resistant breast cancer cells with or without RSL3 for 12 h. (**D**,**E**) Representative live-cell fluorescence images of cells co-transfected with RFP-LC3 and either the empty GFP vector (Vector) or the GFP-STAT3 fusion plasmid (*STAT3* OE). Cells were treated with RSL3 at the indicated concentration (MDA-MB-453: 12 µM; MCF7: 8 µM; HCC1937: 10 µM) for12 h. Images show RFP-LC3 (red) and GFP/GFP-STAT3 (green) signals, acquired without antibody staining. The Vector group expresses free GFP, while the STAT3 OE group expresses the GFP-STAT3 fusion protein (**D**) (Scale bar, 20 µm). The ratio of RFP-LC3 puncta per cell was quantified using ImageJ Plus software (**E**). Data were shown as mean ± SD, and statistical significance was assessed by one-way ANOVA with Bonferroni post-test (*n* > 3, **** *p* < 0.0001). Original images can be found at Supplementary Materials.

To further investigate whether RSL3-mediated autophagy during ferroptosis was related to STAT3, we co-treated PARPi-resistant cells with RSL3 and ferroptosis inhibitors such as Fer-1 and Lip-1, and apoptosis inhibitor Z-VAD-FMK (Z-VAD), or BafA1. We observed that Fer-1 and Lip-1 but not Z-VAD or BafA1 markedly reversed RSL3-induced reduction in STAT3 protein levels, although BafA1 obviously increased p62 and LC3-II/β-actin ratio (Figure 5A), suggesting that STAT3 contributes to the RSL3-induced autophagy during ferroptosis. To verify this possibility, we constructed *STAT3-*overexpressing cell lines and performed Western blot analysis to confirm the overexpression efficiency (Figure 5B). To further confirm that RSL3 promotes STAT3 degradation even under overexpression conditions, we have performed Western blot experiments to assess STAT3 protein levels in *STAT3*-overexpressing cells treated with RSL3. The new data clearly show that RSL3 still promotes STAT3 degradation even under STAT3 overexpression conditions (Figure 5C). Here, we used the STAT3 antibody from Cell Signaling Technology (CST #12640S), which recognizes both the α (79 kDa) and β (86 kDa) isoforms of STAT3. This is a well-documented characteristic of this antibody and reflects the natural expression of STAT3 isoforms in cells. This further supports the conclusion that RSL3 promotes STAT3 degradation via the ubiquitin–proteasome pathway. We then co-transfected cells with RFP-LC3 and either the empty GFP vector (Vector) or the GFP-STAT3 plasmid (*STAT3* OE). Since STAT3 overexpression attenuates RSL3 sensitivity, we applied higher doses of RSL3 (MDA-MB-453: 12 µM; MCF7: 8 µM; HCC1937: 10 µM) to ensure observable autophagic and apoptotic responses. As shown in Figure 5D,E, RSL3 significantly increased LC3 puncta in Vector control cells, and the increased effect was reversed by overexpression of STAT3. The observed reduction in GFP fluorescence upon RSL3 treatment is likely due to general cytotoxic effects or global protein degradation under RSL3-induced stress, rather than specific STAT3 degradation. This is consistent with our previous findings that RSL3 induces apoptosis and cellular stress in Figure 2E and our previous study [16]. Taken together, RSL3-induced autophagy during ferroptosis in PARPi-resistant cells at least partially via decreasing STAT3 protein levels.

### 3.6. Overexpression of STAT3 Decreases RSL3-Induced Apoptosis

**Figure 6:**
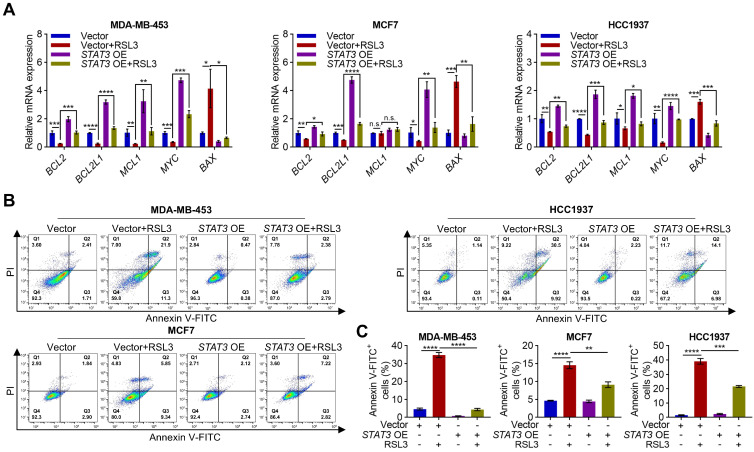
RSL3 induces apoptosis through STAT3. (**A**) RT-qPCR analysis of the indicated apoptosis-related genes in vector or *STAT3*-overexpressing cells treated with a serial dose of RSL3 (MDA-MB-453: 12 µM; MCF7: 8 µM; HCC1937: 10 µM) for 12 h. Data were shown as mean ± SD, and statistical significance was assessed by two-way ANOVA with Tukey’s post-test (*n* = 3, * *p* < 0.05, ** *p* < 0.01, *** *p* <0.001, **** *p* < 0.0001, or n.s., not significant by unpaired Student’s *t* test). (**B**,**C**) Vector and *STAT3*-overexpressing cells were treated with or without RSL3 (MDA-MB-453: 12 µM; MCF7: 8 µM; HCC1937: 10 µM) for 12 h and stained with annexin V-FITC/PI using flow cytometry (**B**). The apoptosis rate was plotted followed by statistical analysis (**C**). Data were shown as mean ± SD, and statistical significance was assessed by one-way ANOVA with Bonferroni post-test (*n* = 3, ** *p* < 0.01, *** *p* <0.001, **** *p* < 0.0001).

How is STAT3 involved in RSL3-induced apoptosis? It is activated in various tumors and pro motes tumor growth, metastasis, and anti-apoptosis [34]. To assess the role of STAT3 in RSL3-induced apoptosis, we used elevated RSL3 doses in STAT3-overexpressing cells to compensate for the conferred resistance. We then measured anti-apoptotic and pro-apoptotic factors mRNA levels in *STAT3-*overexpressing PARPi-resistant cells with or without RSL3. We found that overexpression of STAT3 also significantly attenuated RSL3-induced changes in the expression of anti-apoptotic genes *BCL2*, *BCL2L1*, and *MYC* as well as pro-apoptotic gene *BAX* (Figure 6A). Furthermore, STAT3 overexpression significantly reduced RSL3-induced apoptosis cells (Figure 6B,C). These findings indicate that RSL3 promotes apoptosis through STAT3 in PARPi-resistant breast cancer cells.

## 4. Discussion

**Figure 7:**
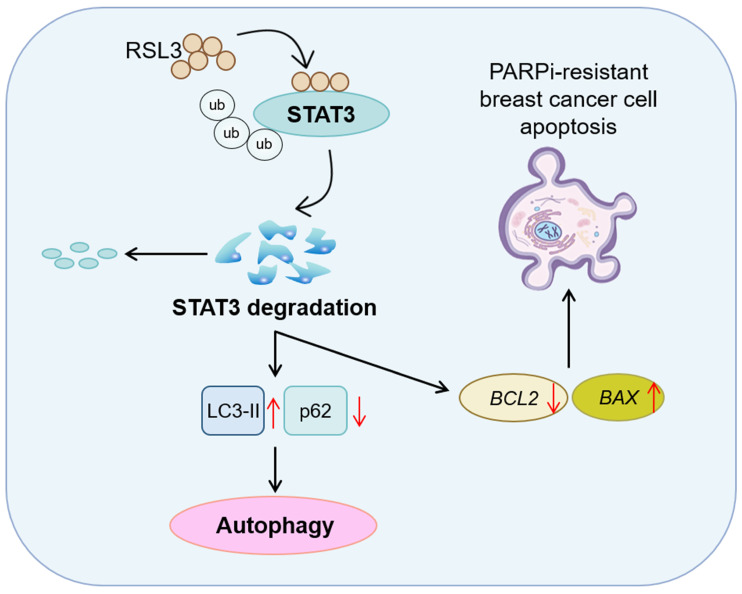
A schematic illustration of the proposed pro-autophagy and pro-apoptosis of RSL3 in PARPi-resistant breast cancer cells. RSL3 targets STAT3 to degrade its protein levels through the ubiquitination pathway. STAT3 degradation leads to both autophagy induction and transcriptional regulation of apoptotic genes, which collectively contribute to apoptosis in PARPi-resistant breast cancer cells.

In this study, we demonstrate that the ferroptosis inducer RSL3 triggers STAT3 ubiquitination and subsequent degradation, ultimately enhancing autophagy and apoptosis in PARPi-resistant breast cancer cells (Figure 7). These findings not only elucidate a novel mechanism of RSL3 action but also highlight STAT3 as a critical mediator of autophagy and apoptosis in the context of PARPi resistance.

STAT3, an oxidative stress-responsive transcription factor, has been increasingly implicated in stress-induced ferroptosis and apoptosis, partly through its regulation of lysosomal membrane permeability [21,24,25]. Previous work has shown that STAT3 promotes lysosomal cell death during ferroptosis by modulating cathepsin B expression and release [21]. The therapeutic relevance of targeting STAT3 is further underscored by studies with the small-molecule degrader SD-36, which triggers selective STAT3 ubiquitination and proteasomal degradation, leading to potent apoptosis induction via caspase-3/7 activation and PARP1 cleavage [25]. Our own prior research demonstrated that ciclopirox promotes autophagic cell death through ubiquitin–proteasome-mediated STAT3 degradation [27]. Building on this foundation, the present study reveals that RSL3 similarly facilitates STAT3 ubiquitination, thereby driving autophagy and apoptosis in PARPi-resistant cells. Moreover, STAT3 overexpression reversed RSL3-induced autophagy and apoptosis, underscoring its pivotal role in this way. This is consistent with studies showing that STAT3 degradation via the ubiquitin–proteasome pathway can trigger autophagy and apoptosis [25,27]. Collectively, these observations position STAT3 as a central signaling hub that interconnects autophagy, apoptosis, and ferroptosis, highlighting its broad therapeutic potential in cancer treatment.

Autophagy plays a double-edged role in cell survival and death. While basal autophagy maintains cellular homeostasis [35], uncontrolled, elongated, or intensive autophagy can promote programmed cell death [28,36], including apoptosis, necroptosis [37], and ferroptosis [38]. Our data show that RSL3-induced autophagy is characterized by elevated LC3-II levels and reduced p62, consistent with robust autophagic activation. The attenuation of RSL3-induced apoptosis upon BafA1-mediated autophagy inhibition confirms the functional contribution of autophagy to cell death in this model. These findings are in line with previous reports that autophagy facilitates ferroptosis through the degradation of GPX4 and ferritin [39,40], and that lysosomal dysfunction enhances ferroptotic sensitivity [20,41]. Notably, erastin and RSL3 have been shown to downregulate GPX4 and FTH1 protein levels, an effect reversible by BafA1, which also inhibits Fe^2+^ accumulation [23]. Together, these results suggest that RSL3 may amplify autophagic activity during ferroptosis, contributing to its cytotoxic effects.

The crosstalk among ferroptosis, autophagy, and apoptosis is complex and highly context-dependent. Our data suggest that RSL3-induced ferroptosis initiates a signaling cascade, leading to STAT3 degradation, which in turn promotes autophagy and apoptosis. This interpretation is supported by the observation and ferroptosis inhibitors, but not inhibitors of apoptosis or autophagy, prevented STAT3 degradation. Cytoplasmic STAT3 has previously been identified as a negative regulator of autophagy, and its degradation—as exemplified by SD-36—can promote apoptosis [25]. Moreover, STAT3 inhibition has been reported to enhance p53-mediated apoptosis [42], a pathway known to be critical in DNA damage-induced cell death [43]. Upon activation, cytoplasmic p53 downregulates Bcl-2 and promotes mitochondrial accumulation of Bax, leading to cytochrome c release, caspase-3 activation, and apoptosis [44,45,46]. Our results suggest that STAT3 is critically involved in RSL3-induced apoptosis, possibly through interactions with these established apoptotic regulators.

Recent studies have revealed that RSL3 enhances ROS-mediated apoptosis in myelodysplastic syndrome [15]. In addition, our preliminary data indicate that RSL3 augments PARP1-mediated apoptotic functions through distinct mechanisms during ferroptosis [16]. These observations reinforce the multifaceted role of RSL3 in engaging multiple cell death pathways and support its potential utility in overcoming therapy-resistant cancers. Several limitations of this study should be acknowledged. First, the specific E3 ligase responsible for RSL3-induced STAT3 ubiquitination remains unidentified. Second, the in vivo relevance of these findings needs further validation. Finally, the potential off-target effects of RSL3 and its efficacy in combination with other therapeutic agents warrant further additional investigation.

## 5. Conclusions

In conclusion, our study uncovers a previously unrecognized mechanism by which RSL3 promotes STAT3 ubiquitination and degradation, leading to autophagy and apoptosis in PARPi-resistant breast cancer cells. These findings not only advance our understanding of PARPi resistance mechanisms but also provides a rationale for targeting the STAT3–autophagy axis as a therapeutic strategy. Future studies exploring RSL3 in combination with PARPi or other targeted agents may offer new hope for patients with resistant disease.

## Data Availability

This data that support the findings of this study are available upon reasonable request from the corresponding author.

## Supplementary Materials

The following supporting information can be downloaded at: https://www.mdpi.com/article/10.3390/biom15121749/s1, Table S1: Primers for gene amplification in the process of RT-qPCR. File S1: WB original figures.

## Abbreviations

The following abbreviations are used in this manuscript:

BafA1	Bafilomycin A1
CQ	Chloroquine
CTRP	Cancer Therapeutics Response Portal
CHX	Cycloheximide
CETSA	Cellular thermal shift assay
DAPI	4′,6-diamidino-2-phenylindole
Fer-1	Ferrostatin-1
GPX4	Glutathione peroxidase 4
GDSC	Genomics of Drug Sensitivity in Cancer
HRP	Horseradish peroxidase
Lip-1	Liproxstatin-1
MG132	Z-Leu-Leu-Leu-al
ROS	Reactive oxygen species
RT-qPCR	Real-time quantitative polymerase chain reaction
STAT3	Signal transducers and activators of transcription 3
PARP	poly(ADP-ribose) polymerase
PARPi	PARP inhibitors
PINK1	PTEN-induced putative kinase 1
PVDF	Polyvinylidene fluoride
3-MA	3-Methyladenine
Z-VAD	Z-VAD-FMK

## References

[1] Bray F., Laversanne M., Sung H., Ferlay J., Siegel R.L., Soerjomataram I., Jemal A. (2024). Global cancer statistics 2022: GLOBOCAN estimates of incidence and mortality worldwide for 36 cancers in 185 countries. CA Cancer J. Clin..

[2] Tew W.P., Lacchetti C., Ellis A., Maxian K., Banerjee S., Bookman M., Jones M.B., Lee J.-M., Lheureux S., Liu J.F. (2020). PARP Inhibitors in the Management of Ovarian Cancer: ASCO Guideline. J. Clin. Oncol..

[3] Tung N.M., Robson M.E., Ventz S., Santa-Maria C.A., Nanda R., Marcom P.K., Shah P.D., Ballinger T.J., Yang E.S., Vinayak S. (2020). TBCRC 048: Phase II Study of Olaparib for Metastatic Breast Cancer and Mutations in Homologous Recombination-Related Genes. J. Clin. Oncol..

[4] Reiss K.A., Mick R., O’Hara M.H., Teitelbaum U., Karasic T.B., Schneider C., Cowden S., Southwell T., Romeo J., Izgur N. (2021). Phase II Study of Maintenance Rucaparib in Patients With Platinum-Sensitive Advanced Pancreatic Cancer and a Pathogenic Germline or Somatic Variant in BRCA1, BRCA2, or PALB2. J. Clin. Oncol..

[5] Fallah J., Xu J., Weinstock C., Gao X., Heiss B.L., Maguire W.F., Chang E., Agrawal S., Tang S., Amiri-Kordestani L. (2024). Efficacy of Poly(ADP-ribose) Polymerase Inhibitors by Individual Genes in Homologous Recombination Repair Gene-Mutated Metastatic Castration-Resistant Prostate Cancer: A US Food and Drug Administration Pooled Analysis. J. Clin. Oncol..

[6] Lord C.J., Ashworth A. (2017). PARP inhibitors: Synthetic lethality in the clinic. Science.

[7] Langelier M.-F., Planck J.L., Roy S., Pascal J.M. (2012). Structural basis for DNA damage-dependent poly(ADP-ribosyl)ation by human PARP-1. Science.

[8] Li H., Liu Z.-Y., Wu N., Chen Y.-C., Cheng Q., Wang J. (2020). PARP inhibitor resistance: The underlying mechanisms and clinical implications. Mol. Cancer.

[9] Liang C., Zhang X., Yang M., Dong X. (2019). Recent Progress in Ferroptosis Inducers for Cancer Therapy. Adv. Mater..

[10] Chen H., Qi Q., Wu N., Wang Y., Feng Q., Jin R., Jiang L. (2022). Aspirin promotes RSL3-induced ferroptosis by suppressing mTOR/SREBP-1/SCD1-mediated lipogenesis in PIK3CA-mutant colorectal cancer. Redox Biol..

[11] Mahoney-Sánchez L., Bouchaoui H., Ayton S., Devos D., Duce J.A., Devedjian J.-C. (2020). Ferroptosis and its potential role in the physiopathology of Parkinson’s Disease. Prog. Neurobiol..

[12] Deng F., Zhao B.-C., Yang X., Lin Z.-B., Sun Q.-S., Wang Y.-F., Yan Z.-Z., Liu W.-F., Li C., Hu J.-J. (2021). The gut microbiota metabolite capsiate promotes Gpx4 expression by activating TRPV1 to inhibit intestinal ischemia reperfusion-induced ferroptosis. Gut Microbes.

[13] Li Y., Feng D., Wang Z., Zhao Y., Sun R., Tian D., Liu D., Zhang F., Ning S., Yao J. (2019). Ischemia-induced ACSL4 activation contributes to ferroptosis-mediated tissue injury in intestinal ischemia/reperfusion. Cell Death Differ..

[14] Dixon S.J., Lemberg K.M., Lamprecht M.R., Skouta R., Zaitsev E.M., Gleason C.E., Patel D.N., Bauer A.J., Cantley A.M., Yang W.S. (2012). Ferroptosis: An iron-dependent form of nonapoptotic cell death. Cell.

[15] Liu L., Yang C., Zhu L., Wang Y., Zheng F., Liang L., Cao P., Liu J., Han X., Zhang J. (2024). RSL3 enhances ROS-mediated cell apoptosis of myelodysplastic syndrome cells through MYB/Bcl-2 signaling pathway. Cell Death Dis..

[16] Chen D., Xie F., Mo Y., Qin D., Zheng B., Chen L. (2025). RSL3 promotes PARP1 apoptotic functions by distinct mechanisms during ferroptosis. Cell Mol. Biol. Lett..

[17] Filomeni G., De Zio D., Cecconi F. (2015). Oxidative stress and autophagy: The clash between damage and metabolic needs. Cell Death Differ..

[18] Park E., Chung S.W. (2019). ROS-mediated autophagy increases intracellular iron levels and ferroptosis by ferritin and transferrin receptor regulation. Cell Death Dis..

[19] Gao M., Monian P., Pan Q., Zhang W., Xiang J., Jiang X. (2016). Ferroptosis is an autophagic cell death process. Cell Res..

[20] Gao H., Bai Y., Jia Y., Zhao Y., Kang R., Tang D., Dai E. (2018). Ferroptosis is a lysosomal cell death process. Biochem. Biophys. Res. Commun..

[21] Zhou B., Liu J., Kang R., Klionsky D.J., Kroemer G., Tang D. (2020). Ferroptosis is a type of autophagy-dependent cell death. Semin. Cancer Biol..

[22] Yu F., Zhang Q., Liu H., Liu J., Yang S., Luo X., Liu W., Zheng H., Liu Q., Cui Y. (2022). Dynamic O-GlcNAcylation coordinates ferritinophagy and mitophagy to activate ferroptosis. Cell Discov..

[23] Liu J., Liu Y., Wang Y., Li C., Xie Y., Klionsky D.J., Kang R., Tang D. (2023). TMEM164 is a new determinant of autophagy-dependent ferroptosis. Autophagy.

[24] Lei Z., Yu J., Wu Y., Shen J., Lin S., Xue W., Mao C., Tang R., Sun H., Qi X. (2024). CD1d protects against hepatocyte apoptosis in non-alcoholic steatohepatitis. J. Hepatol..

[25] Bai L., Zhou H., Xu R., Zhao Y., Chinnaswamy K., McEachern D., Chen J., Yang C.-Y., Liu Z., Wang M. (2019). A Potent and Selective Small-Molecule Degrader of STAT3 Achieves Complete Tumor Regression In Vivo. Cancer Cell.

[26] Shen S., Niso-Santano M., Adjemian S., Takehara T., Malik S.A., Minoux H., Souquere S., Mariño G., Lachkar S., Senovilla L. (2012). Cytoplasmic STAT3 represses autophagy by inhibiting PKR activity. Mol. Cell.

[27] Chen L., Chen D., Li J., He L., Chen T., Song D., Shan S., Wang J., Lu X., Lu B. (2022). Ciclopirox drives growth arrest and autophagic cell death through STAT3 in gastric cancer cells. Cell Death Dis..

[28] Chen L., Chen D., Pan Y., Mo Y., Lai B., Chen H., Zhang D.-W., Xia X.-D. (2024). Inhibition of mitochondrial OMA1 ameliorates osteosarcoma tumorigenesis. Cell Death Dis..

[29] Wang X., Shi W., Li M., Xin Y., Jiang X. (2024). RSL3 sensitizes glioma cells to ionizing radiation by suppressing TGM2-dependent DNA damage repair and epithelial-mesenchymal transition. Redox Biol..

[30] Ireno I.C., Wiehe R.S., Stahl A.I., Hampp S., Aydin S., Troester M.A., Selivanova G., Wiesmüller L. (2014). Modulation of the poly (ADP-ribose) polymerase inhibitor response and DNA recombination in breast cancer cells by drugs affecting endogenous wild-type p53. Carcinogenesis.

[31] Schröder-Heurich B., Bogdanova N., Wieland B., Xie X., Noskowicz M., Park-Simon T.-W., Hillemanns P., Christiansen H., Dörk T. (2014). Functional deficiency of NBN, the Nijmegen breakage syndrome protein, in a p.R215W mutant breast cancer cell line. BMC Cancer.

[32] Guo E., Ishii Y., Mueller J., Srivatsan A., Gahman T., Putnam C.D., Wang J.Y.J., Kolodner R.D. (2020). FEN1 endonuclease as a therapeutic target for human cancers with defects in homologous recombination. Proc. Natl. Acad. Sci. USA.

[33] Kroemer G., Levine B. (2008). Autophagic cell death: The story of a misnomer. Nat. Rev. Mol. Cell Biol..

[34] Al Zaid Siddiquee K., Turkson J. (2008). STAT3 as a target for inducing apoptosis in solid and hematological tumors. Cell Res..

[35] Kroemer G., Mariño G., Levine B. (2010). Autophagy and the integrated stress response. Mol. Cell.

[36] Gump J.M., Staskiewicz L., Morgan M.J., Bamberg A., Riches D.W.H., Thorburn A. (2014). Autophagy variation within a cell population determines cell fate through selective degradation of Fap-1. Nat. Cell Biol..

[37] He W., Wang Q., Srinivasan B., Xu J., Padilla M.T., Li Z., Wang X., Liu Y., Gou X., Shen H.-M. (2014). A JNK-mediated autophagy pathway that triggers c-IAP degradation and necroptosis for anticancer chemotherapy. Oncogene.

[38] Hou W., Xie Y., Song X., Sun X., Lotze M.T., Zeh H.J., Kang R., Tang D. (2016). Autophagy promotes ferroptosis by degradation of ferritin. Autophagy.

[39] Wu Z., Geng Y., Lu X., Shi Y., Wu G., Zhang M., Shan B., Pan H., Yuan J. (2019). Chaperone-mediated autophagy is involved in the execution of ferroptosis. Proc. Natl. Acad. Sci. USA.

[40] Liu Y., Wang Y., Liu J., Kang R., Tang D. (2021). Interplay between MTOR and GPX4 signaling modulates autophagy-dependent ferroptotic cancer cell death. Cancer Gene Ther..

[41] Chen L., Ning J., Linghu L., Tang J., Liu N., Long Y., Sun J., Lv C., Shi Y., Tao T. (2025). USP13 facilitates a ferroptosis-to-autophagy switch by activation of the NFE2L2/NRF2-SQSTM1/p62-KEAP1 axis dependent on the KRAS signaling pathway. Autophagy.

[42] Liu Y., Lv J., Liu J., Liang X., Jin X., Xie J., Zhang L., Chen D., Fiskesund R., Tang K. (2018). STAT3/p53 pathway activation disrupts IFN-β-induced dormancy in tumor-repopulating cells. J. Clin. Investig..

[43] Marión R.M., Strati K., Li H., Murga M., Blanco R., Ortega S., Fernandez-Capetillo O., Serrano M., Blasco M.A. (2009). A p53-mediated DNA damage response limits reprogramming to ensure iPS cell genomic integrity. Nature.

[44] Chipuk J.E., Bouchier-Hayes L., Kuwana T., Newmeyer D.D., Green D.R. (2005). PUMA couples the nuclear and cytoplasmic proapoptotic function of p53. Science.

[45] Salvador-Gallego R., Mund M., Cosentino K., Schneider J., Unsay J., Schraermeyer U., Engelhardt J., Ries J., García-Sáez A.J. (2016). Bax assembly into rings and arcs in apoptotic mitochondria is linked to membrane pores. EMBO J..

[46] Wu C.-C., Lee S., Malladi S., Chen M.-D., Mastrandrea N.J., Zhang Z., Bratton S.B. (2016). The Apaf-1 apoptosome induces formation of caspase-9 homo- and heterodimers with distinct activities. Nat. Commun..

