# Supplementary material for: RSL3 Promotes STAT3 Ubiquitination to Induce Autophagy and Apoptosis in PARPi-Resistant Breast Cancer Cells
Source: Biomolecules. 2025 Dec 18;15(12):1749. doi: 10.3390/biom15121749 (PMC12730874; doi:10.3390/biom15121749)
Supplement: Supplementary file 1 [file biomolecules-15-01749-s001.zip › biomolecules-3956235-supplementary table.pdf]

**Supplementary Table 1 Primers for gene amplification in the process of RT-qPCR.**

| Target gene | The sequence of primers from 5’ to 3’ |
|---|---|
| Human-*BCL2* | Forward: GGTGGGGTCATGTGTGTGG<br>Reverse: CGGTTCAGGTACTCAGTCATCC |
| Human-*BCL2L1* | Forward: GAGCTGGTGGTTGACTTTCTC<br>Reverse: TCCATCTCCGATTCAGTCCCT |
| Human-*MCL1* | Forward: AGCACAGAGCCTCGCCTTTG<br>Reverse: AAGCCGGCCTTGCACATG |
| Human-*MYC* | Forward: GGCTCCTGGCAAAAGGTCA<br>Reverse: CTGCGTAGTTGTGCTGATGT |
| Human-*BAX* | Forward: CCCGAGAGGTCTTTTTCCGAG<br>Reverse: CCAGCCCATGATGGTTCTGAT |
| Human-*STAT3* | Forward: CGGAGAAGCATCGTGAGTGAGC<br>Reverse: GTTGCCGCCTCTTCCAGTCAG |
| Human-*β-ACTIN* | Forward: AGCACAGAGCCTCGCCTTTG<br>Reverse: AAGCCGGCCTTGCACATG |
| Human-*GAPDH* | Forward: GGAGCGAGATCCCTCCAAAAT<br>Reverse: GGCTGTTGTCATACTTCTCATGG |
