# Supplementary figures and images for: RSL3 Promotes STAT3 Ubiquitination to Induce Autophagy and Apoptosis in PARPi-Resistant Breast Cancer Cells
Source: Biomolecules. 2025 Dec 18;15(12):1749. doi: 10.3390/biom15121749 (PMC12730874; doi:10.3390/biom15121749)

Fig. 2C

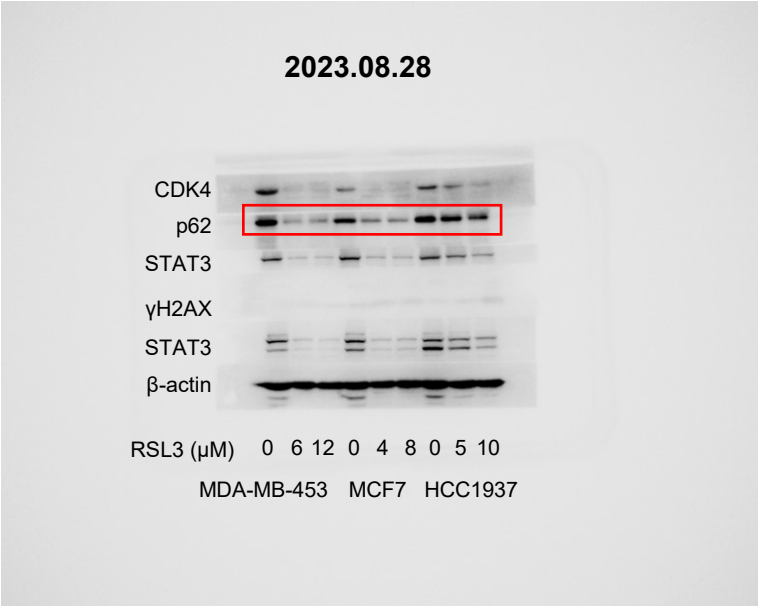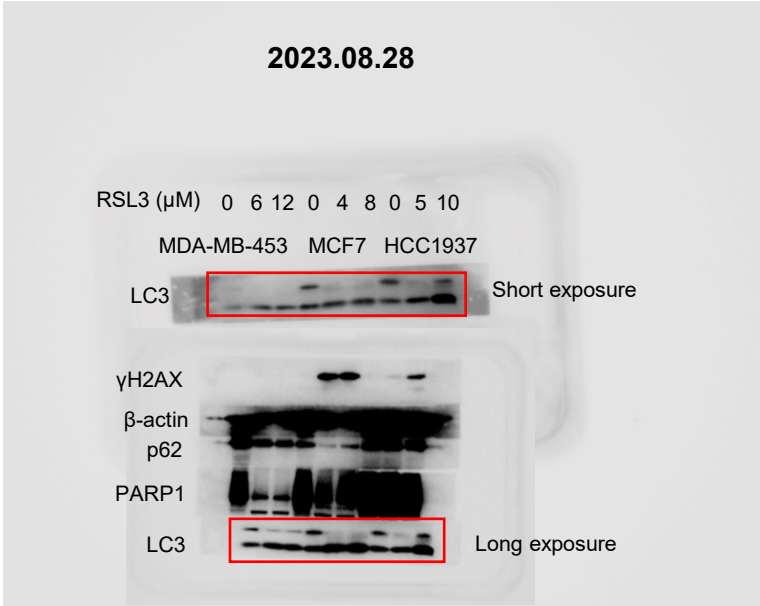

Fig. 2D

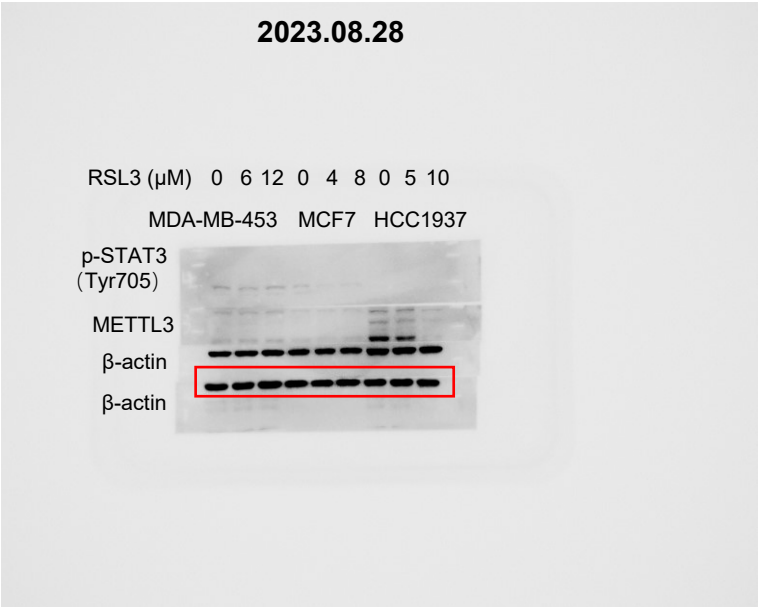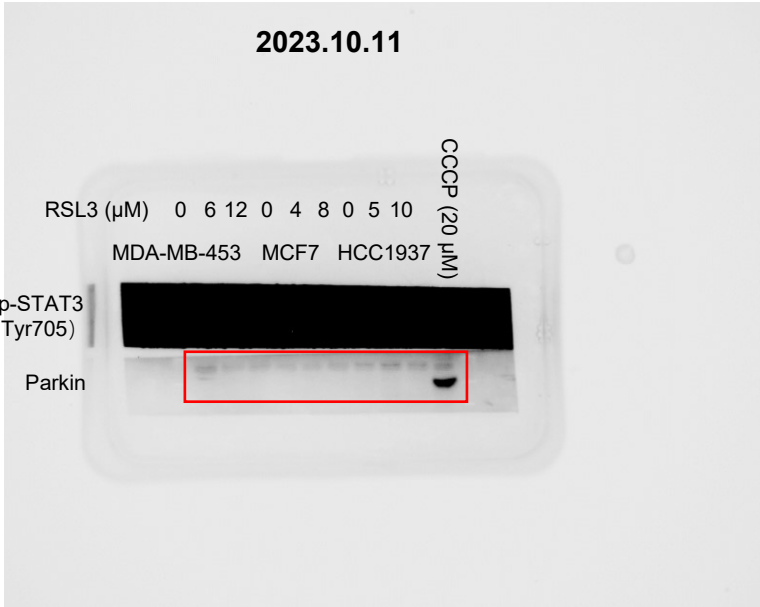

Fig. 3B

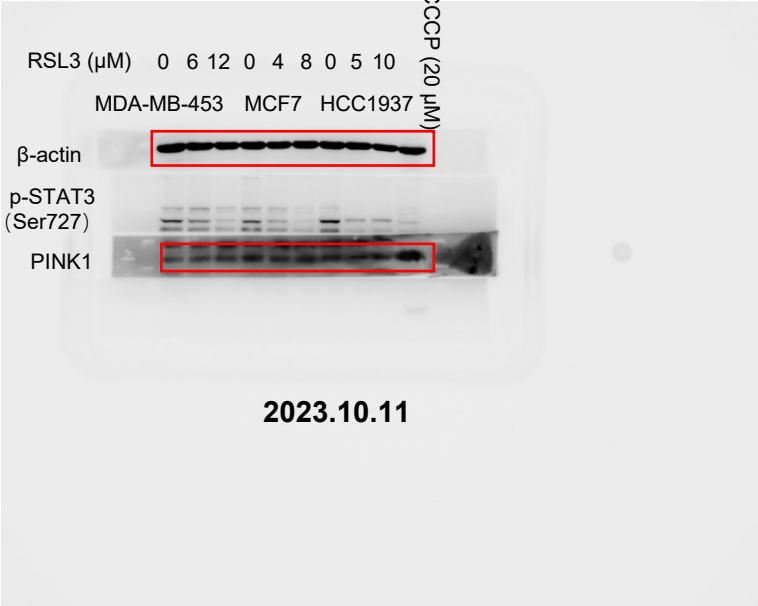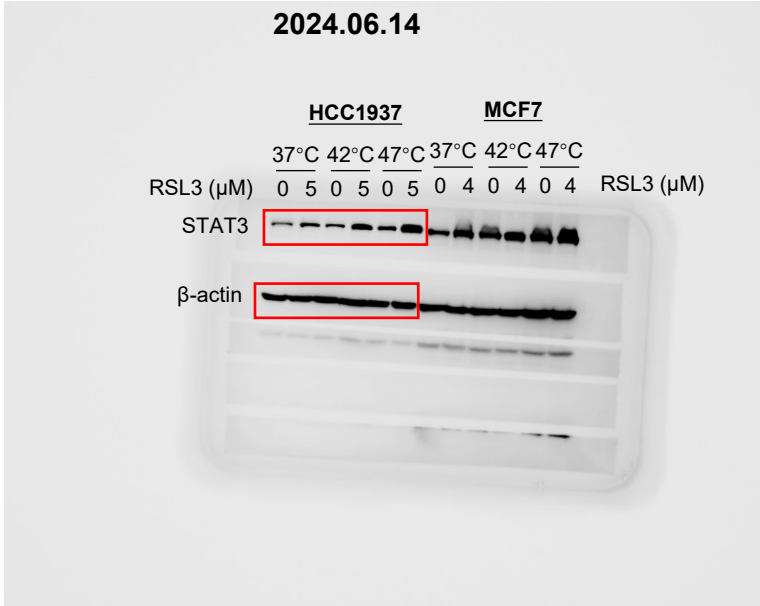

Fig. 3B

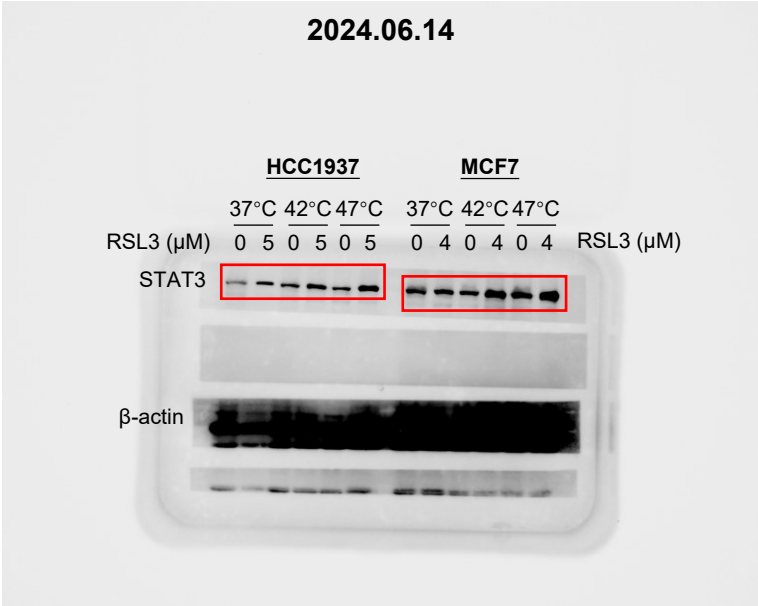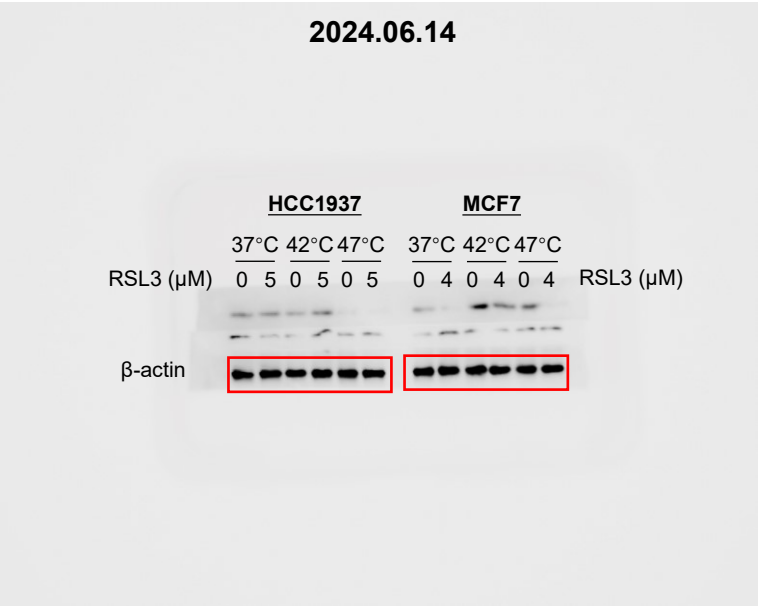

Fig. 3C

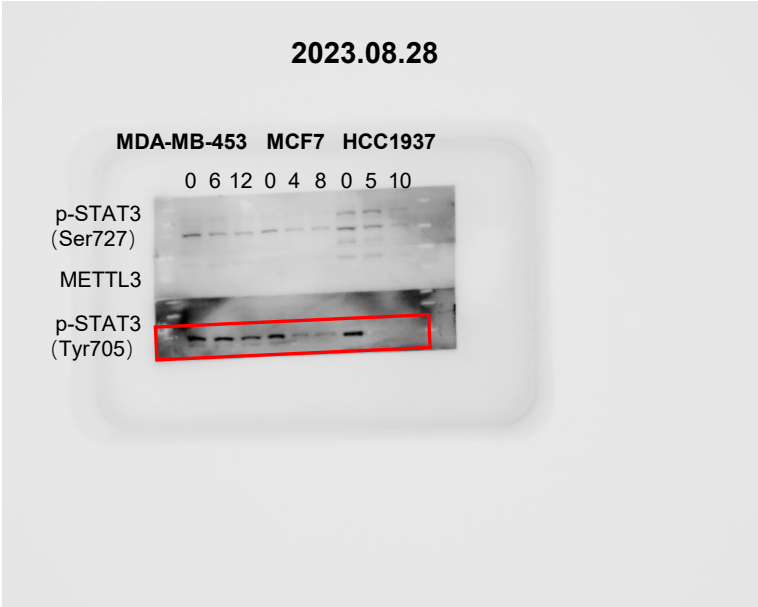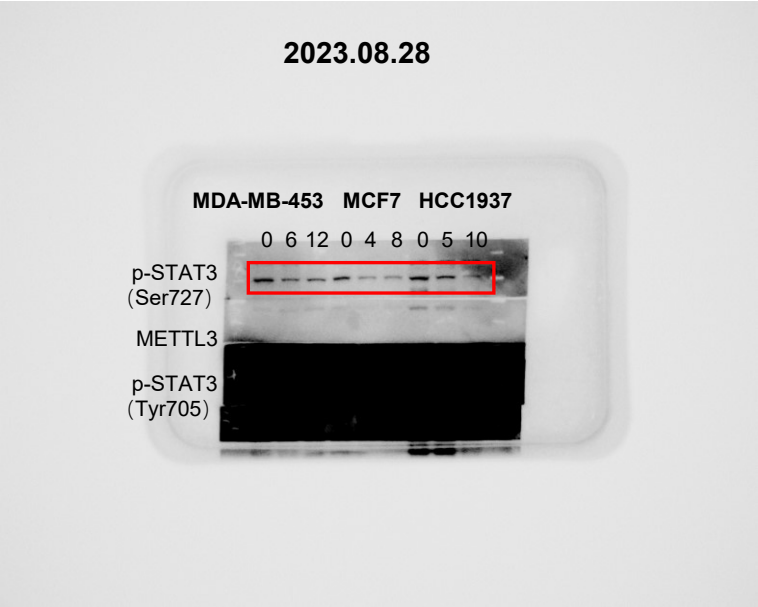

Fig. 4A

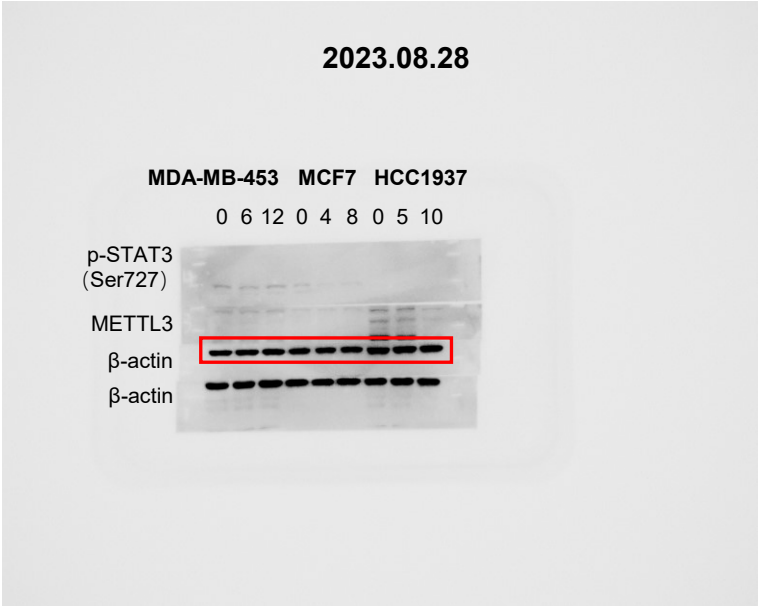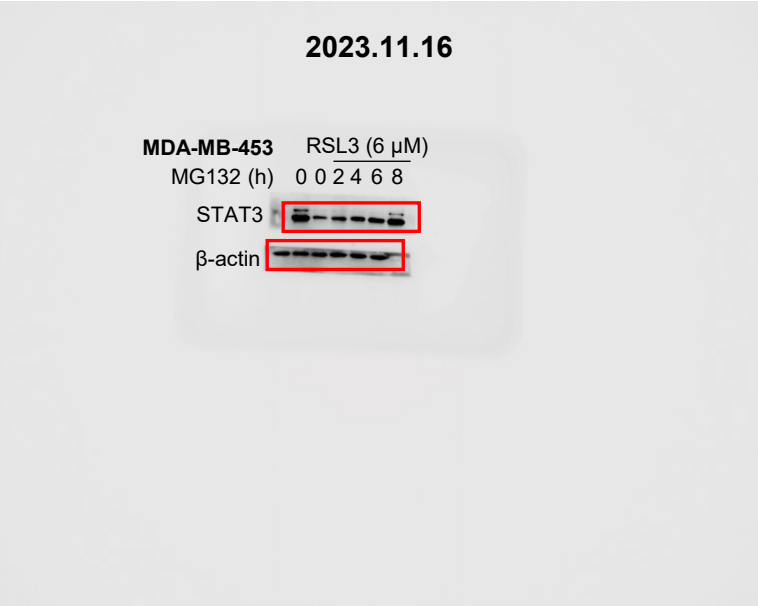

Fig. 4A

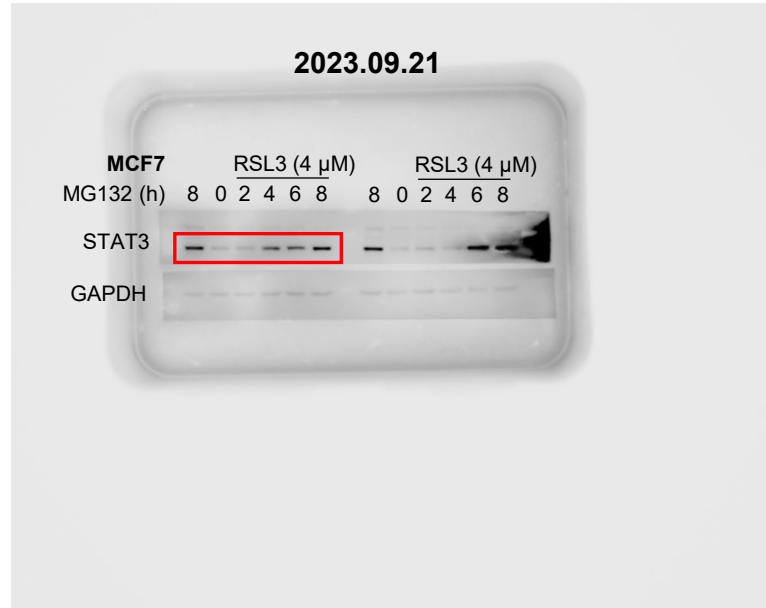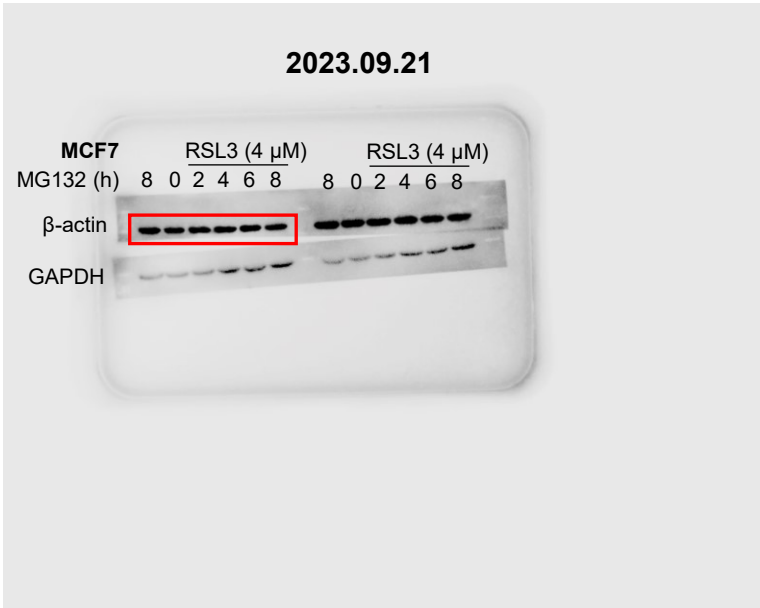

Fig. 4B

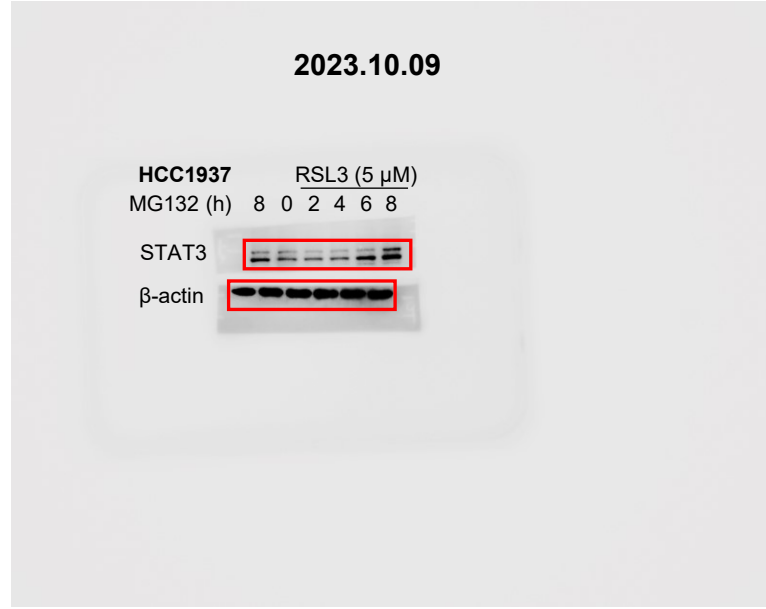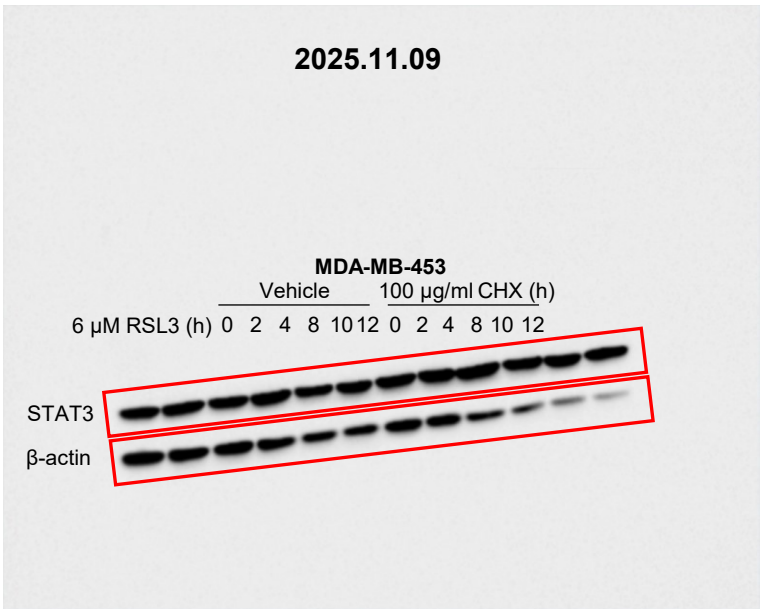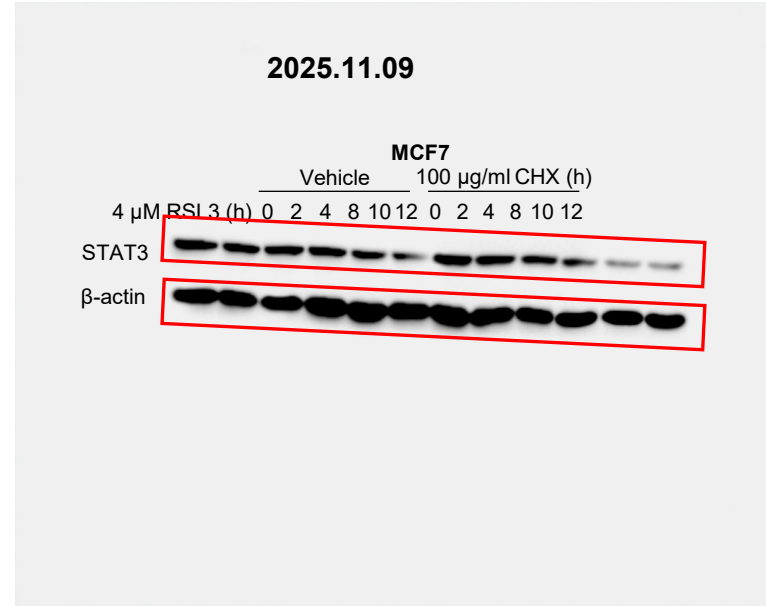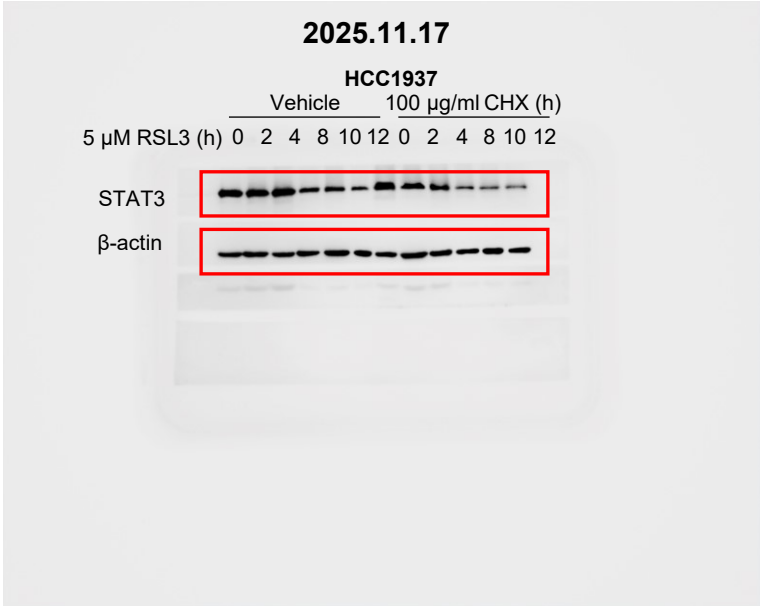

Fig. 4C

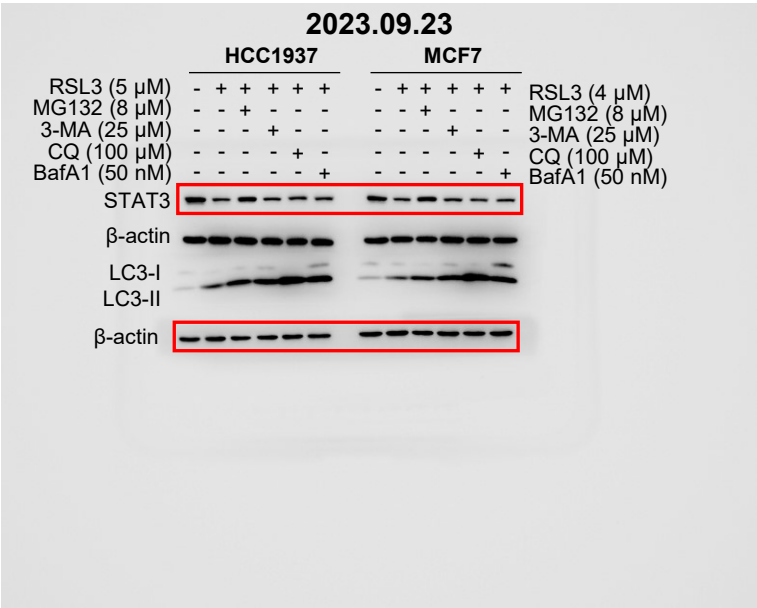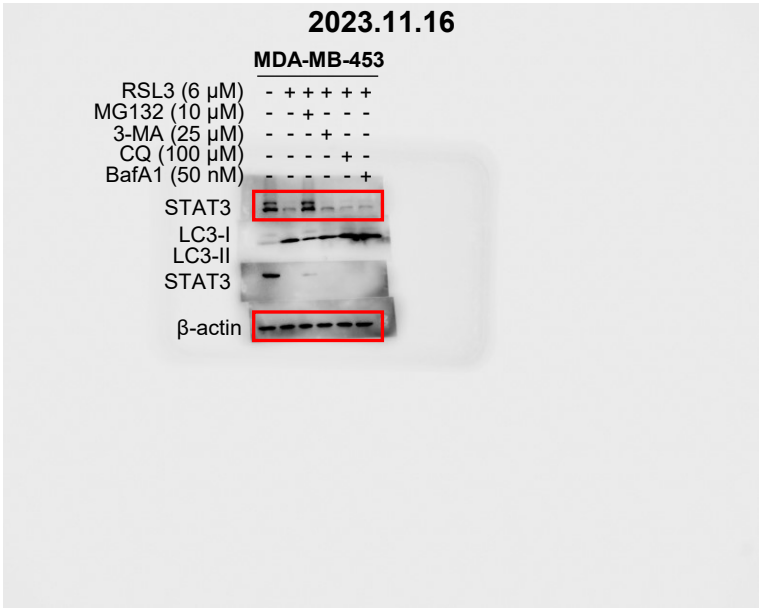

Fig. 4D

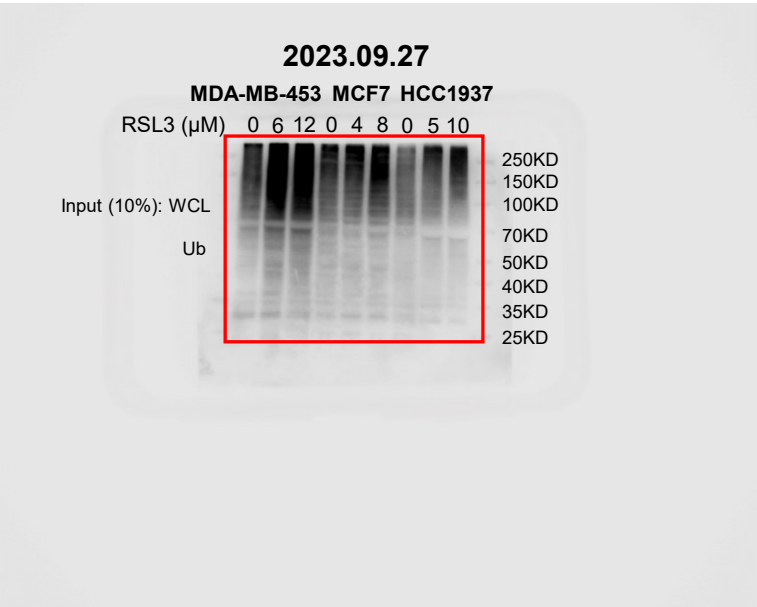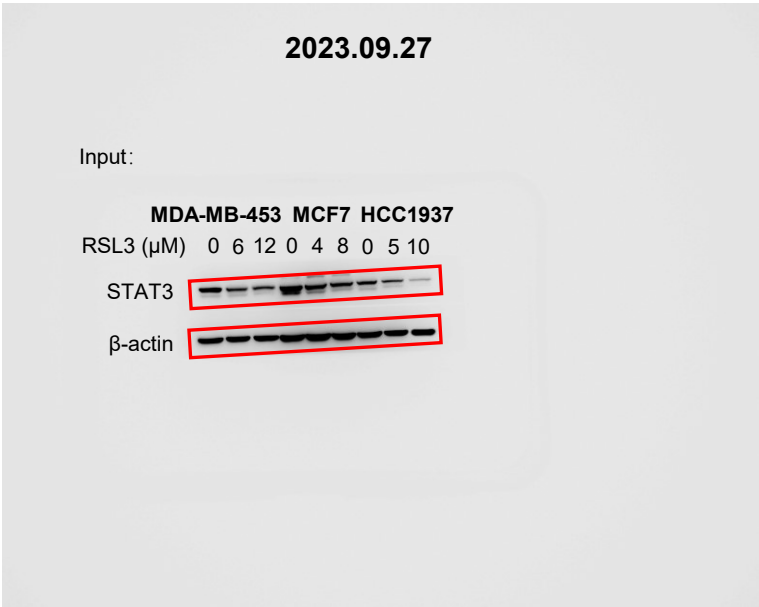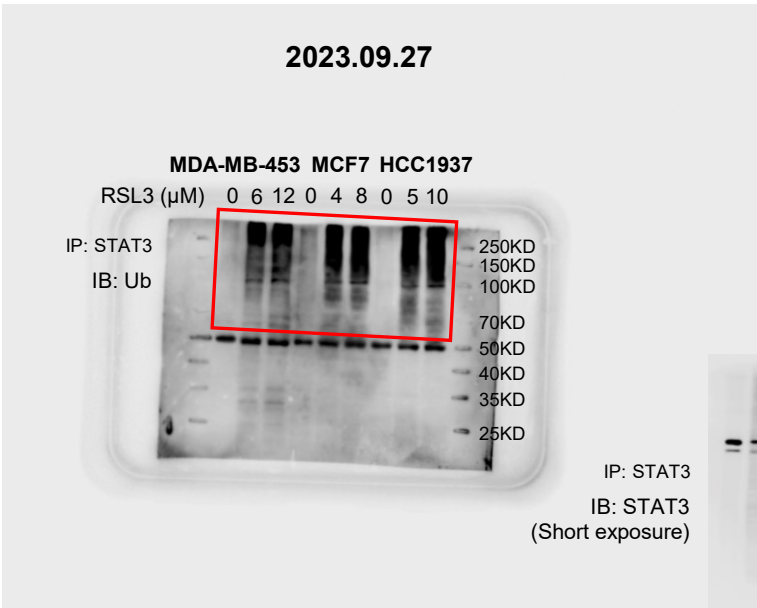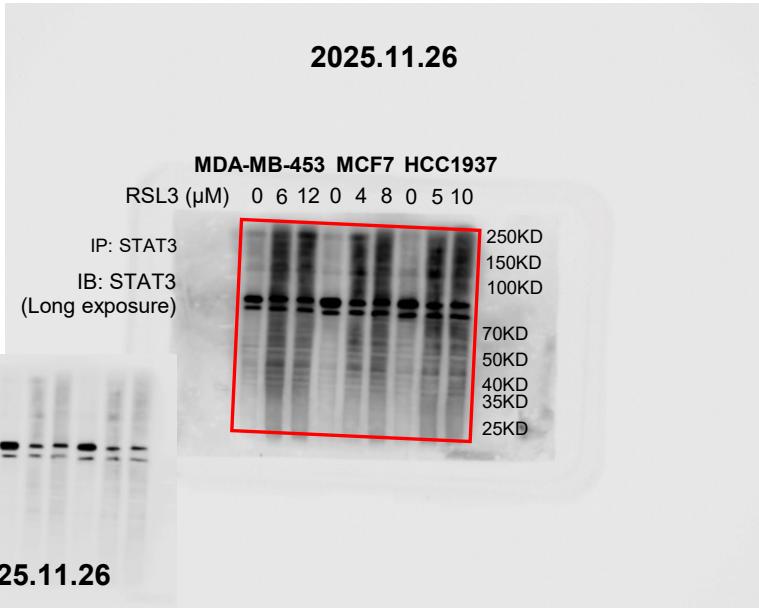

Fig. 4E

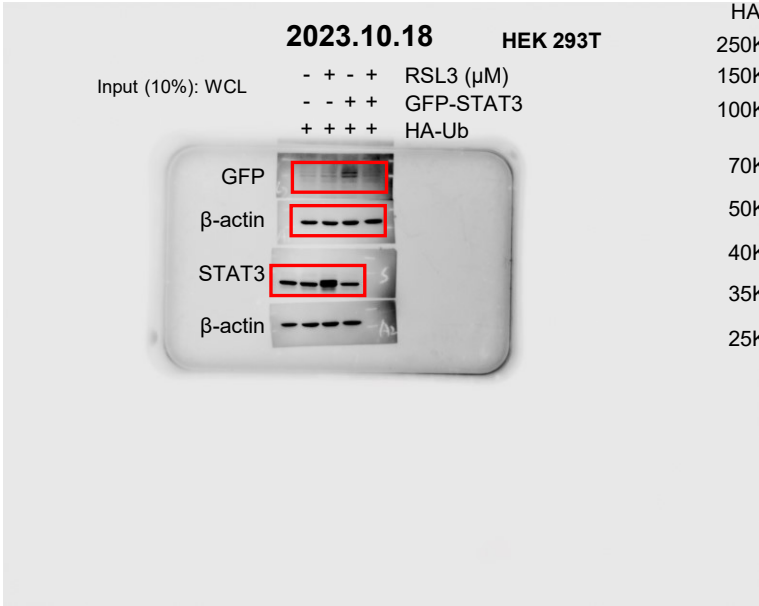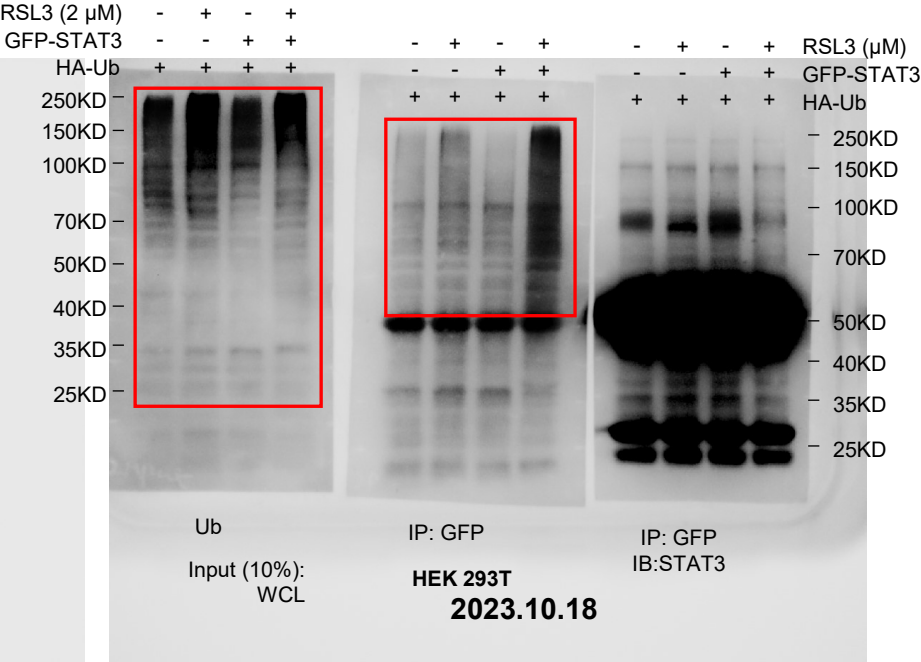

Fig. 5A

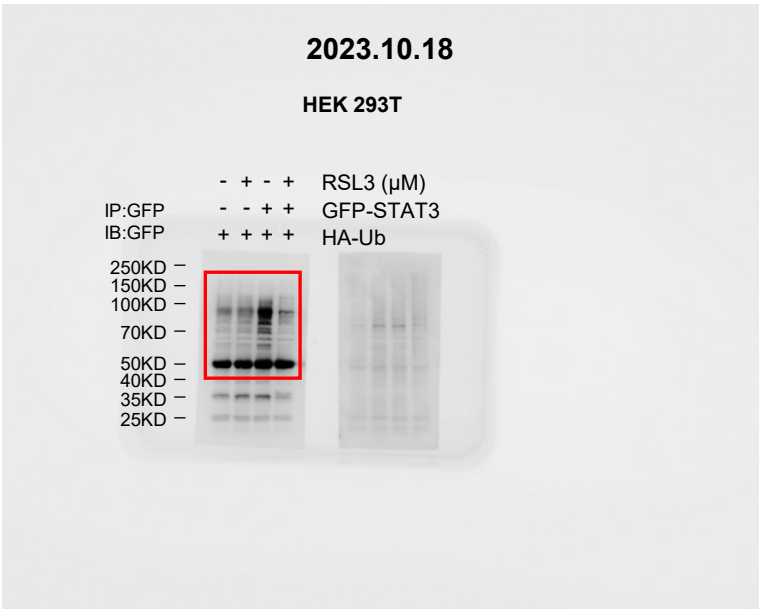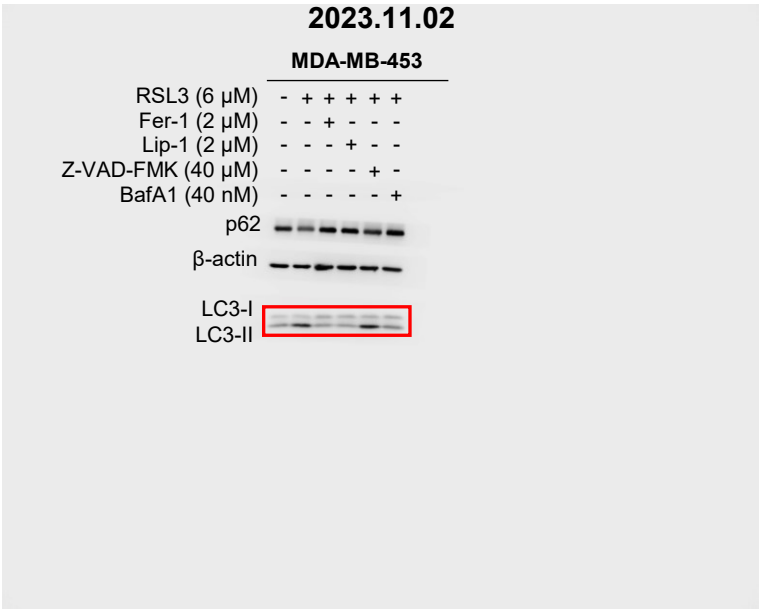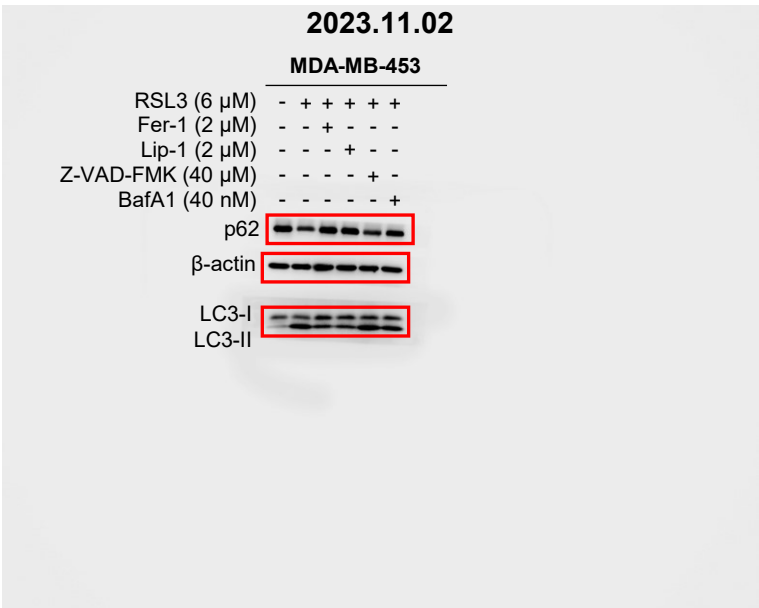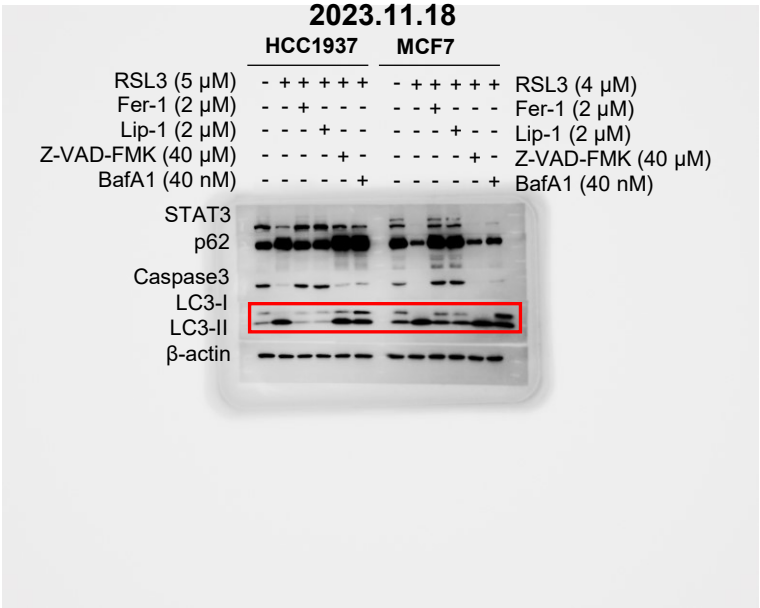

Fig. 5A

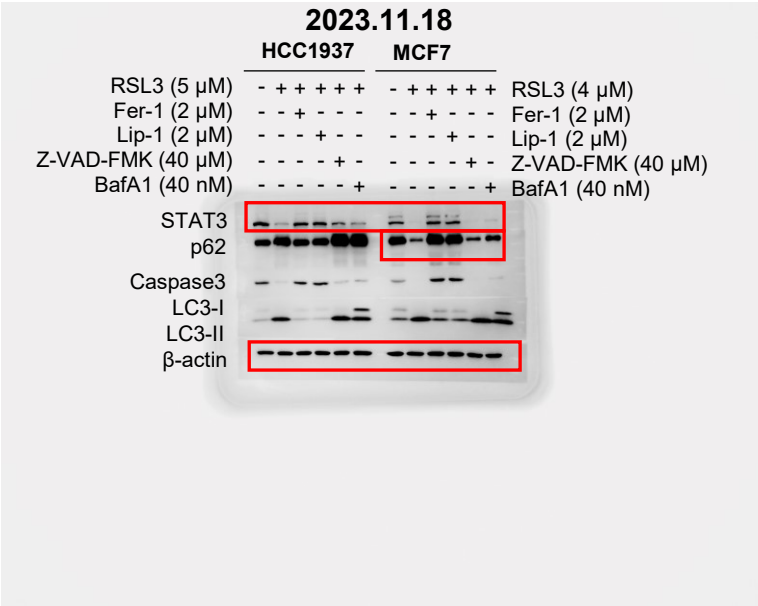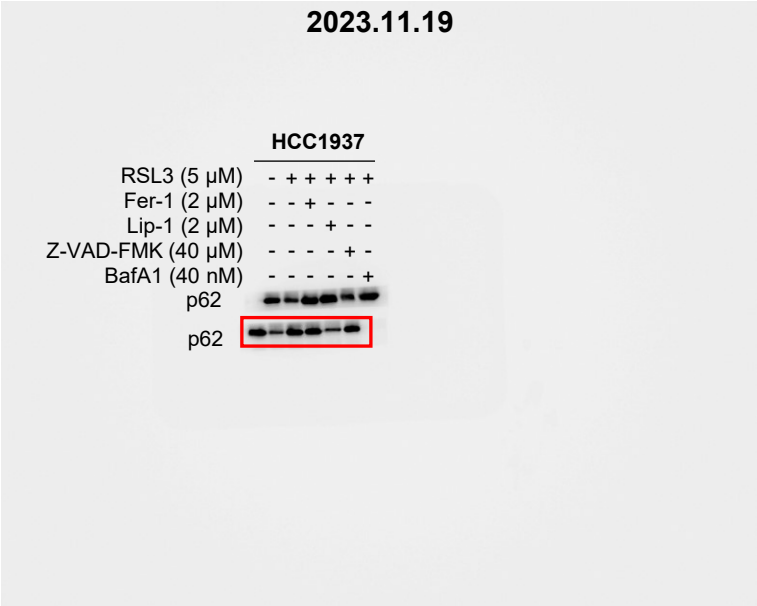

Fig. 5B

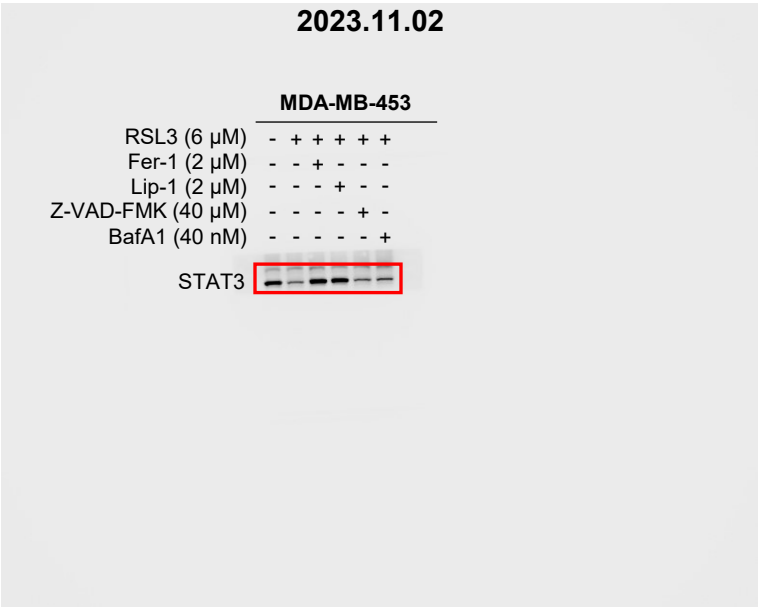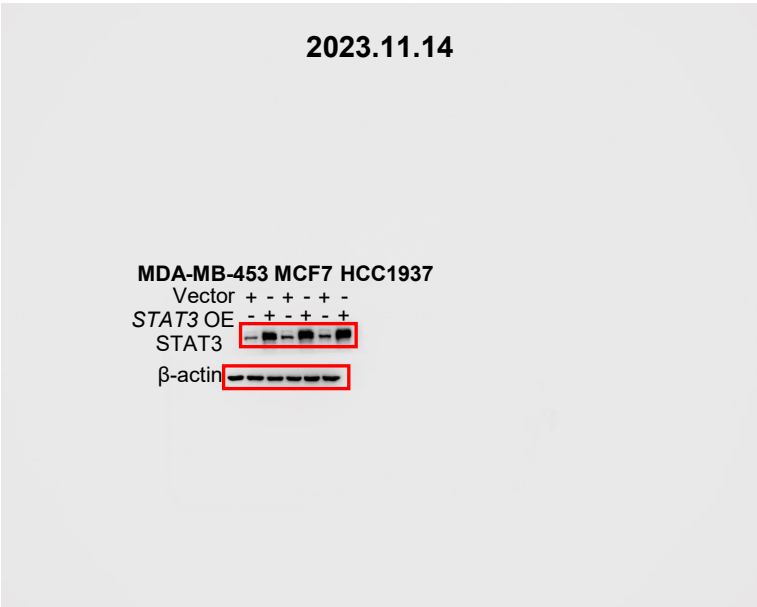

Fig. 5C

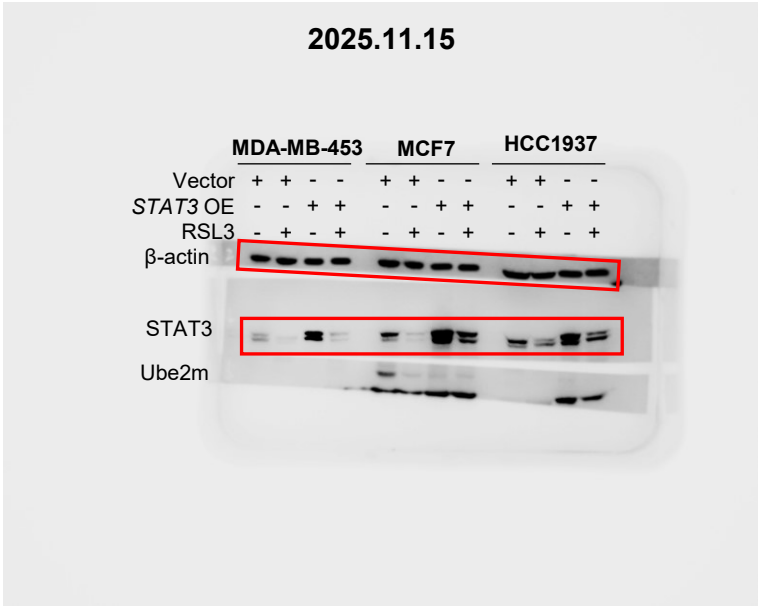

Supplement: Supplementary file 1 [file biomolecules-15-01749-s001.zip › biomolecules-3956235-original-images.pdf]
